# Switch Catalysis To Deliver Multi‐Block Polyesters from Mixtures of Propene Oxide, Lactide, and Phthalic Anhydride

**DOI:** 10.1002/anie.201810245

**Published:** 2018-11-26

**Authors:** Tim Stößer, Daniel Mulryan, Charlotte K. Williams

**Affiliations:** ^1^ Department of Chemistry Oxford University Chemical Research Laboratory 12 Mansfield Road Oxford OX1 3TA UK

**Keywords:** aluminium salen catalysts, block polyesters, ring-opening copolymerization, ring-opening polymerization, switch catalysis

## Abstract

Switchable polymerisation catalysis enables block polymer sequence selectivity from monomer mixtures, resulting in the formation of multiblock polyesters. The aluminium salphen catalyst switches between two different polymerisation mechanisms and selectively enchains mixtures of commercially available monomers: lactide, phthalic anhydride, and propene oxide. Sequential monomer mixture additions yield multi‐block polyesters featuring 3, 7, 11, 15, 19, 23, and 27 blocks. The unparalleled catalytic selectivity can be used to access completely new multi‐block polyesters relevant for future applications.

The selective transformation of reagent mixtures into well‐defined and useful products is a grand challenge for sustainable catalysis.^1^ Such multicomponent catalysis could simplify process chemistry and obviate the energy, time, and labour currently needed for intermediary isolation, purification, and protection/deprotection reaction steps.^1^, ^2^ For polymerisations, it may also be used to control monomer sequence and polymer microstructures.^2^ This work reports a catalytic route to block‐sequence‐selective multi‐block polymers from monomer mixtures. Most block polymers studied to date have simple AB or ABA structures, in part owing to difficulties in accessing more sophisticated block patterns.^3^ To deliver block polymers featuring structurally diverse repeat units, it is important to understand how to combine different polymerisation mechanisms and monomer classes. Such a strategy requires a means to “switch” a single catalyst between different polymerisation cycles.^4^

In 2014, our group reported the first “switch catalysis” by using a dizinc catalyst and mixtures of cyclohexene oxide/CO_2_/ϵ‐caprolactone to produce poly(carbonate‐*b*‐ester)s with single types of block structures.^5^ Kinetic and DFT studies indicate that the catalysis is controlled by both thermodynamic (linkage stability) and kinetic factors (transition‐state barriers).^6^ The dizinc catalysts were also selective for ABA triblock polyester production, from mixtures of cyclohexene oxide/anhydrides/ϵ‐decalactone,^7^ or ABCBA pentablock polymers, from cyclohexene oxide/CO_2_/anhydride/ϵ‐decalactone mixtures.^8^ Recently, chain extension of some of these ABA triblock polymers yielded materials that were effective thermoplastic elastomers, rigid plastics, or shape‐memory materials, depending on the block compositions and phase‐separated microstructures.^9^ Subsequently, other dizinc(II) and Cr^III^ catalysts were identified as effective switch catalysts to make block polymers (Scheme 1).^10^

**Scheme 1:**
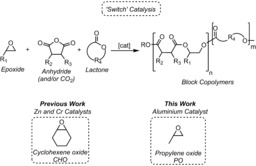
Switch catalysis and work covered herein.

Here, the goal was to apply switch catalysis to monomers that are commercially available and already used at large scale in polymer manufacturing: namely propene oxide (9 Mt/annum worldwide production),^11^ lactide (ca. 1 Mt/annum worldwide),^12^ and phthalic anhydride (4.5 Mt/annum; Figure 1).^13^ These monomers are currently used to produce polyether polyols (PPO), biodegradable plastics (PLA), and cross‐linked resins (PA); thus far, there is no precedent for selective polymerisation of mixtures of them. In any switchable catalytic polymerisation using three different monomers, a number of products could form (see the Supporting Information, Schemes S1 and S2). For example, not all monomers may react, leading to contaminated mixtures of homopolymer(s); the resulting copolymers could show random or gradient structures, particularly if only kinetic factors control enchainment. Considering only diad sequences, five different linkages are feasible: three esters (PA‐PO or LA‐LA or LA‐PA), an ether (PO‐PO), and an ester‐ether (LA‐PO); as more monomers are enchained, the number of possible sequences increases. Additionally, other catalysts exposed to mixtures of PO/LA yielded either only isotactic PLA or formed 3,6‐dimethyl‐1,4‐dioxan‐2‐one (lactone).^14^ Other side reactions could be PO ring‐opening polymerisation (ROP) to polyethers^15^ and/or unselective ring‐opening copolymerisation (ROCOP) to form poly(ether‐ester)s.^16^ Overall, these particular monomers bring additional complexities and potential side reactions compared to previous studies, and these problems must be overcome for successful switch catalysis.

**Figure 1:**
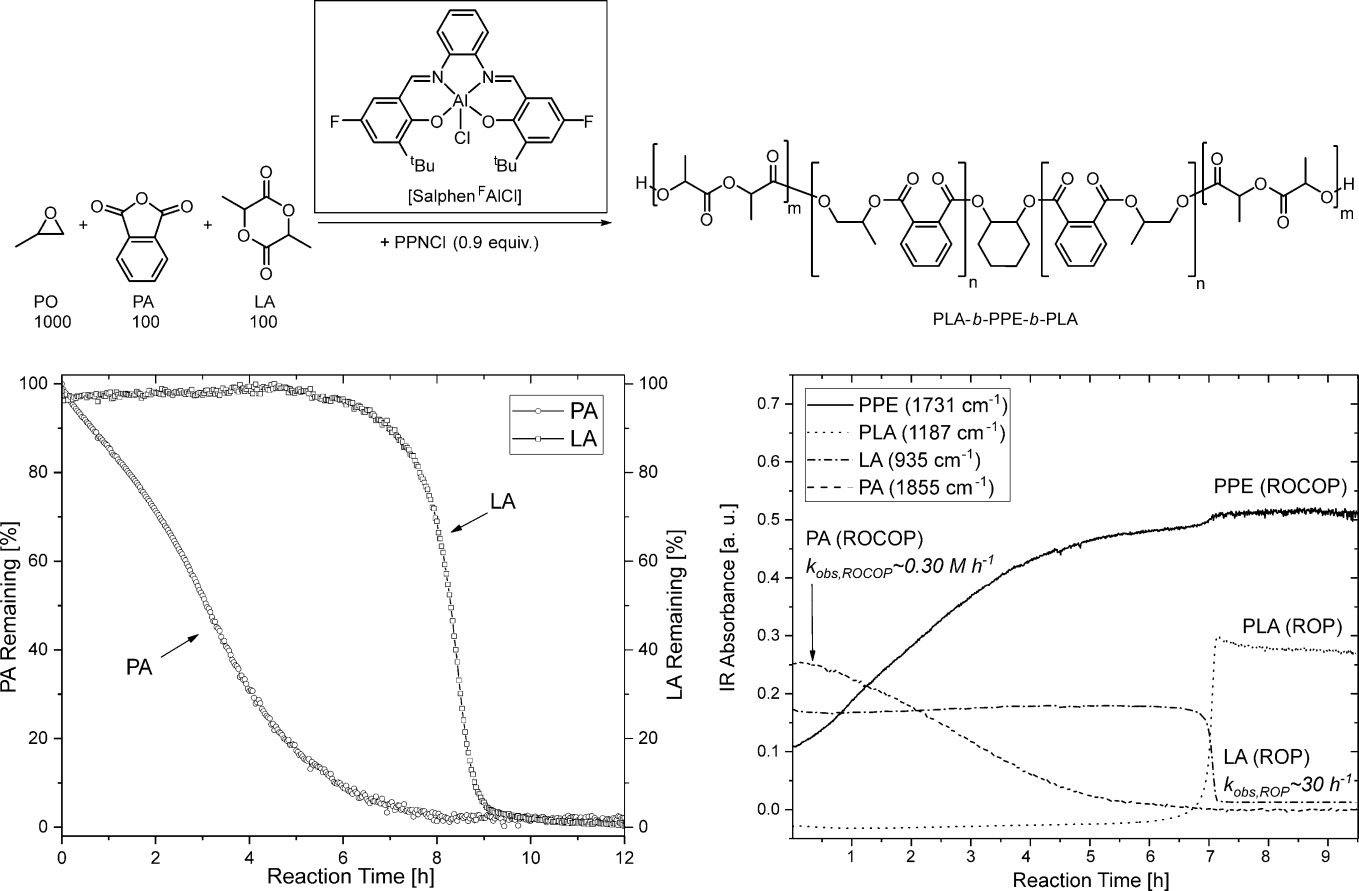
Switch catalysis with PO, PA, LA (top). The in situ IR plot is shown on the left; the in situ ^1^H NMR monitoring is shown on the right. Reaction conditions: [Salphen^F^AlCl]/[PPNCl]/[CHD]/[PA]/[LA]/[PO]=1:0.8:10:100:100:1000, 60 °C (IR) and 1:0.8:10:50:50:150, 0.75 m in CDCl_3_ (NMR).

As a starting point, PA/PO ROCOP and LA ROP were each separately attempted using a commercial aluminium salen chloride catalyst system ([SalcyAlCl]; Tables S1 and S2). In both cases, although in situ spectroscopic analysis confirmed switch catalysis, the resulting polyesters showed high dispersity values, indicating transesterification. Such transesterification side reactions are a known difficulty in alternating polymerisations using PO.1c, 17 Recently, Coates and co‐workers reported a modified Al‐salen catalyst featuring electron‐withdrawing fluorine substituents ([Salphen^F^AlCl], Figure 1), which showed significantly reduced transesterification in the ROCOP of PO/anhydrides.^18^ Inspired by this work, we investigated [Salphen^F^AlCl] for the one‐pot combination of PO, PA, and LA, with the addition of cyclohexane diol as a chain‐transfer agent (Figure 1, top). Under these conditions, the catalysis was highly selective for ABA triblock polyesters. The resulting polyesters showed predictable molar masses and low dispersities (*Ð*≈1.2) even at full monomer conversions (>99 % PA, >95 % LA). Importantly, the GPC traces remained narrow even for 12 h (in contact with catalyst) after reaction completion (Figure S1). To unambiguously identify the polymer product, a series of analytical techniques were applied according to the recommendations previously described for switch catalysis.4c

The one‐pot polymerisation of PO/PA/LA was monitored by in situ IR spectroscopy (Figure 1, right). PO/PA ROCOP occurred first, as indicated by the decrease in intensity of a band assigned to PA and the coincident increase in the poly(propylene phthalate) (PPE) band. Only after high conversions (>95 %) of PA were achieved did LA ROP occur. This is a key criterion for the synthesis of well‐defined block polyesters from switch catalysis. In terms of TOFs, ROCOP was slightly slower (TOF≈15 h^−1^) then ROP (TOF≈400 h^−1^); further kinetic analysis revealed a zeroth order rate constant for ROCOP with *k*_PO/PA‐ROCOP_=8.20±0.02×10^−5^ m s^−1^, and a first‐order rate constant for ROP of *k*_LA‐ROP_=8.05±0.50×10^−3^ s^−1^ (Figure S2 and Table S3). It should be noted that higher rates than previously reported may result from running these reactions in sealed vials to create an overpressure in PO (boiling point: 34 °C vs. reaction temperature: 60 °C).^19^ Selective monomer incorporation was also confirmed by in situ ^1^H NMR monitoring (Figure 1, left), which showed no evidence for PO homopolymerisation at any point during the reaction (Figure S3). There was no stereoselectivity in the resulting PLA, as evidenced by broad resonances attributed to atactic PLA at *δ*=5.15 ppm (Figure S3).

The reaction was monitored by GPC by removal of aliquots after completion of ROCOP (97 % PA, <5 % LA) and after ROP (99 % PA, >95 % LA). The molar mass increased between the two reaction stages, as expected for a block polyester; for a mixture of homopolymers, two separate peaks are expected (Figure S4). Moreover, PPE contains a chromophore, which allows analysis of GPC traces by two different detectors, refractive index (RI) and UV (254 nm). The GPC traces obtained from both detectors were overlaid, both before and after the ROP step, which confirmed the attachment of the PLA block to PPE (Figure S4).

The polymerisation was conducted in the presence of excess diol to ensure the formation of telechelic ABA triblock polyesters with hydroxy end groups. These hydroxy end groups were identified using ^31^P{^1^H} NMR spectroscopy, after reaction with 2‐chloro‐4,4,5,5‐tetramethyl‐1,3,2‐dioxaphospholane, and were calibrated using an internal standard (bisphenol A).^20^ The triblock polymer showed only one set of signals, at *δ*=147.16 ppm, identical to the result obtained using pure PLA and different from that with PPE (*δ*=158.56 and 146.29 ppm; Figure S5). Furthermore, the block polymer was investigated using a range of 2D NMR techniques, and a junction unit connecting the two blocks was identified by TOCSY NMR spectroscopy (Figure S18).

^13^C{^1^H} NMR spectra for the triblock copolymers were compared to the corresponding homopolymers and showed characteristic differences (Figure S6): First, the triblock copolymers showed only one set of end group resonances, at *δ*=175.12 ppm; these resonances were the same as for pure PLA, but different to those for PPE (*δ*=167.59 ppm). Second, the block polymers showed two carbonyl resonances at chemical shifts that were identical to those of the homopolymers, at *δ*=168.82 ppm (PPE) and *δ*=169.48 ppm (PLA). This finding confirmed the presence of both blocks, and the lack of intermediary signals suggests any transesterification was at levels below the resolution limit of the technique. Third, the catalyst shows low regioselectivity for PO/PA ROCOP as all regioisomers were observed (*δ*=167.18–166.68 ppm; Figure S7).^21^ This finding also helps rationalise the two slightly separated signals, due to primary and secondary hydroxy end groups, observed in the ^31^P{^1^H} NMR spectrum for PPE.

The block polymer was also analysed by DOSY NMR spectroscopy: Only one diffusion coefficient was detected for all signals, indicating that the blocks are covalently attached to one another (*D*=1.78×10^−8^ m^2^ s^−1^; Figure S8, top). A blend of homopolymers yielded two different diffusion coefficients, confirming that block polyesters are the sole product of this catalysis (*D*_PLA_=7.93×10^−6^ m^2^ s^−1^ and *D*_PPE_=7.28×10^−6^ m^2^ s^−1^; Figure S8).

Finally, the proposed block polyester formed in switch catalysis was compared to a block polyester synthesised through sequential monomer additions. Both polymers showed the same characterisation data when analysed by GPC, IR, and NMR spectroscopy (see the Supporting Information). Overall, all of the analytical tests confirmed the formation of well‐defined ABA triblock polyesters of the form poly(lactide‐*b*‐propylene phthalate‐*b*‐lactide).

The catalysis combines two catalytic cycles: PO/PA ROCOP and LA ROP (Scheme 2). The selectivity observed in the polymer products and the results of the in situ spectroscopy indicate that PO/PA ROCOP proceeds first. The proposed pathway during ROCOP involves PA being incorporated into an aluminium alkoxide intermediate to form an aluminium carboxylate species (*k*_1_). The latter reacts with propylene oxide (*k*_2_) to regenerate the aluminium alkoxide intermediate. The ROCOP reaction shows a zeroth‐order rate dependence on anhydride concentration, suggesting that the Al‐carboxylate attack step is rate‐determining, that is, *k*_1_≫*k*_2_. In situ spectroscopic analysis (both IR and NMR) indicates that the aluminium carboxylate species reacts selectively with PO but not with LA. This key observation is proposed to account for the high selectivity observed in switch catalysis and the clean formation of particular block sequences. LA ROP proceeds through an alkoxide intermediate (*k*_3_). It should be noted that the detailed mechanism of Al‐salen catalysts for anhydride/epoxide ROCOP was recently investigated by Coates, Tolman and co‐workers.^22^ They observed distinctive colour changes during the reaction and ascribed a deep red colour to the formation of an aluminium bis(alkoxide) species, present at the end of ROCOP. This same change in colour was also observed during these switch catalyses, with the colour changing from yellow (ROCOP, where the resting state is an Al‐carboxylate species) to red (ROP, where the resting state is an Al‐alkoxide species) as the reaction proceeded, providing a visual indicator of the stage of the reaction. Whilst a detailed understanding of the intimate reaction mechanism and catalyst speciation is beyond the scope of this work, the proposed mechanism is consistent with the experimental, kinetic, and polymer characterisation data. It should be noted that the term “switching” refers to a change in mechanism between ROCOP and ROP during the catalysis and is controlled by the nature of the metal‐chain end group.

**Scheme 2:**
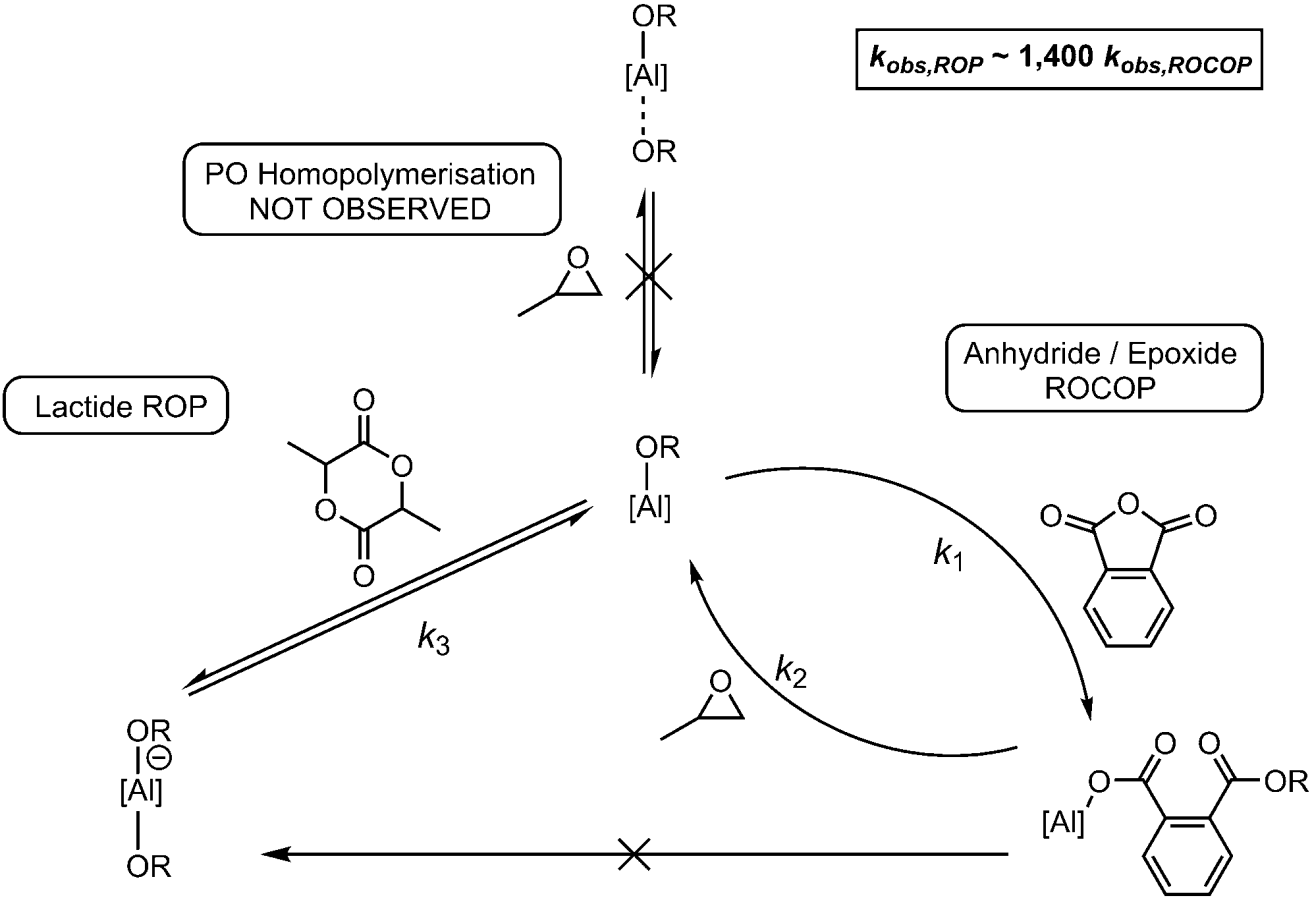
Proposed mechanistic pathways for switch catalysis with [Salphen^F^AlCl].

Sequential monomer addition into a living polymerisation is a useful means to prepare block polymers. This approach has been impressively refined for reactions of acrylate monomers by RAFT, producing multi‐block polyacrylates featuring up to 20 blocks in defined sequence.^23^ For polyesters, related well‐defined multiblocks featuring >10 blocks are very rare, mostly because of synthetic limitations and a lack of sufficiently selective living polymerisation techniques.^24^ Here, the ability to “switch” the [Salphen^F^AlCl] catalyst system was tested by repeatedly adding aliquots containing monomer mixtures of PO/PA/LA to the reaction (Figure 2). The first monomer addition yields ABA triblocks; each subsequent addition adds four more blocks symmetrically to the telechelic chain ends. This sequential monomer mixture addition process was repeated seven times, yielding chains with 7, 11, 15, 19, 23, and eventually an icosikaihepta (27) block polyester.^25^ At the end point, the multi‐block polyester showed a molar mass of 23 500 g mol^−1^ (*Ð*=1.21). After each addition, the crude reaction mixture was analysed by ^1^H NMR spectroscopy, which showed high conversions of all monomers (>99 % PA, >92 % LA; relative to an internal standard) and very high selectivity for ABA pattern enchainment (>99 %; Figure S9). GPC analysis revealed monomodal molar mass distributions, with a continuously increasing molar mass and low dispersities (*Ð*≈1.2) as the additions progressed (Figure 2, left). The GPC RI and UV detection outputs were identical in all cases, indicating that blocks are joined. Plots of molar mass versus block number showed a linear fit to the data and an overall progressive increase in molar mass (Figure 2, right). These multi‐block structures far exceed any previously reported for polyesters and deliver a potential new platform of materials for further structure–property evaluation.

**Figure 2:**
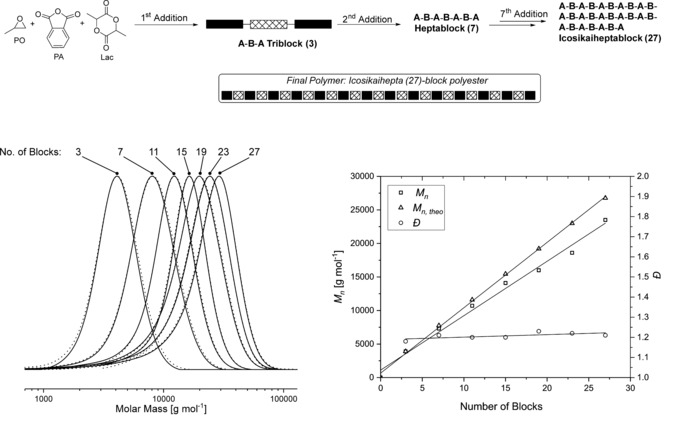
Synthesis of multiblock polymers by multiple monomer addition (top). RI (solid) and UV (dotted) GPC traces recorded after each monomer addition (left) and evolution of *M_n_*, *M*_*n*,*theo*_, and *Ð* (right). Reaction conditions: [Salphen^F^AlCl]/[PPNCl]/[CHD]/[PA]/[LA]/[PO]= 1:0.8:10:100:100:2000, 60 °C; each monomer addition contained 100 equiv of PA and 100 equiv of LA.

Next, the catalytic conditions were evaluated with the goal of reducing catalyst concentration to levels more generally relevant to large‐scale deployment. The study showed successful catalysis at monomer/catalyst loadings from 100:1 to 2000:1. Indeed, the ability to apply 0.05 mol % catalyst loading is unusually low in the context of other anhydride/epoxide ROCOP catalysts.1c, 26 Given the importance of PLA as a commercial bioderived polymer, it was also relevant to consider the potential to source the other monomers from biomass so as to deliver fully bio‐based polyesters.^2^ PO could be produced at sufficient scale from glycerol or carbohydrates and routes to PA from corn stover have been evaluated.^27^ As further proof of potential, switch catalysis was evaluated using mixtures of PO/LA and a bio‐based tricyclic anhydride using [Salphen^F^AlCl] (Figure S10). The reaction resulted in complete monomer conversions (99 %) and, again, very high block sequence selectivity for ABA triblocks (*M_n_*=14 200 g mol^−1^, *Ð*=1.22; Figures S10 and S11). Analysis of this polymer by DSC revealed two glass transition temperatures (*T*_g,1_=45 °C; *T*_g,2_=68 °C), indicating the potential for block microphase separation, which is expected to be relevant for future applications (Figure S12).3a

In conclusion, the highly selective aluminium salphen switchable catalyst polymerised mixtures of propene oxide, lactide, and phthalic anhydride to produce multi‐block polyesters. The catalysis can be controlled to selectively form multi‐block polyesters with 3, 7, 11, 15, 19, 23, and 27 blocks. The use of commercial monomers that are already used industrially and low catalyst loadings shows promise for implementation at scale of this technology. The novel multi‐block polymer structures would be inaccessible or very difficult to prepare by conventional procedures and thus represent a new platform of materials, some of which may be relevant as sustainable polymers. Block polymers show outstanding performances in applications spanning thermoplastic elastomers, toughened plastics, as blend compatibilisers, and in healthcare; such applications may benefit from these new classes of all‐polyester multi‐blocks. These multi‐block structures may also deliver completely new micro‐ or nanostructures, which may confer valuable means to moderate physical‐chemical properties.^3^

## Conflict of interest

The authors declare no conflict of interest.

## Supporting information

As a service to our authors and readers, this journal provides supporting information supplied by the authors. Such materials are peer reviewed and may be re‐organized for online delivery, but are not copy‐edited or typeset. Technical support issues arising from supporting information (other than missing files) should be addressed to the authors.

Supplementary
