# Supplementary material for: Switch Catalysis To Deliver Multi‐Block Polyesters from Mixtures of Propene Oxide, Lactide, and Phthalic Anhydride
Source: Angew Chem Int Ed Engl. 2018 Nov 26;57(51):16893–7. doi: 10.1002/anie.201810245 (PMC6391959; doi:10.1002/anie.201810245)
Supplement: Supplementary file 1 — Supplementary [file ANIE-57-16893-s001.pdf]

Angewandte Chemie
Eine Zeitschrift der Gesellschaft Deutscher Chemiker

Supporting Information

# Switch Catalysis To Deliver Multi-Block Polyesters from Mixtures of Propene Oxide, Lactide, and Phthalic Anhydride

*Tim Stößer, Daniel Mulryan, and Charlotte K. Williams**

anie_201810245_sm_miscellaneous_information.pdf

## List of Figures

Figure S. 1 – One-pot polymerisation of PO/PA/Lactide (top) and GPC analysis at different times (bottom). Conditions: [Salphen$^{F}$AlCl]:[PPNCl]:[CHD]:[PA]:[LA]:[PO] = 1:0.8:10:100:100:1000, 60°C. ........ 9
Figure S. 2 – Kinetics analysis of in situ IR plot shown in Fig. 1. ........ 10
Figure S. 3 – $^{1}$H NNR spectrum of isolated polyester; the region relevant to the formation of polyether between 3.0 and 4.0 ppm is enlarged (polyether should be observed as broad resonances in this area). ........ 11
Figure S. 4 - GPC traces of one-pot polymerisation of PO/PA/LA with [Salphen$^{F}$AlCl]/PPNCl. RI trace is given as a solid, UV trace as a dashed line. Conditions: [Salphen$^{F}$AlCl]:[PPNCl]:[CHD]:[PA]:[LA]:[PO] = 1:0.8:10:100:100:1000, 60°C. ........ 12
Figure S. 5 - $^{31}$P{$^{1}$H} NMR spectra of different polyesters, after the reaction with 2-chloro-4,4,5,5-tetramethyl-1,3,2-dioxaphospholane (bisphenol A present as internal standard). .... 13
Figure S. 6 - Comparison of the $^{13}$C{$^{1}$H} NMR spectra of the block copolymer and the separate polymers obtained from ROP or ROCOP. ........ 14
Figure S. 7 - $^{13}$C{$^{1}$H} NMR spectrum of isolated block copolyester and an illustration of regioselective epoxide ring-opening. ........ 15
Figure S. 8 - DOSY NMR Spectra of block copolymer (A) and blend of homopolymers (B). 16
Figure S. 9 - $^{1}$H NMR spectra of crude reaction mixture after each monomer addition; typical reaction times: 6 hours – 2 days. Conversions are based on mesitylene (6.80 ppm) as internal standard. ........ 17
Figure S. 10 – $^{1}$H NMR spectrum of the one-pot polymerisation of TCA1/PO/LA ........ 18
Figure S. 11 – GPC trace of polymer obtained from TCA1/PO/LA. ........ 19
Figure S. 12 – DSC Thermogram of purified polymer based on PO/PA/LA. ........ 19
Figure S. 13 – IR spectra of monomers and polymers. ........ 20
Figure S. 14 – Extended region of IR spectra for PA and PPE. ........ 20
Figure S. 15 – Extended region of IR spectra for LA and PLA. ........ 21
Figure S. 16 – $^{1}$H NMR spectrum of isolated polymer. ........ 22
Figure S. 17 – $^{13}$C{$^{1}$H} NMR spectrum of isolated polymer. ........ 23
Figure S. 18 – $^{1}$H,$^{1}$H TOCSY NMR spectrum (only region relevant to junction unit shown). 24
Figure S. 19 – $^{1}$H,$^{1}$H TOCSY NMR spectrum. ........ 24
Figure S. 20 – $^{1}$H,$^{1}$H COSY NMR Spectrum of PLA-*b*-PPE-*b*-PLA. ........ 25
Figure S. 21 – GPC Traces of block polyester formed through sequential monomer addition (left) and comparison between polyesters obtained from sequential monomer addition and ‘switch’ catalysis. ........ 26
Figure S. 22 – IR Spectra of polymer obtained from ‘switch’ catalysis and polymer synthesized *via* sequential monomer addition. ........ 26
Figure S. 23 – $^{1}$H NMR Spectra of polymer obtained from ‘switch’ catalysis and polymer synthesized *via* sequential monomer addition. ........ 27
Figure S. 24 – $^{13}$C{$^{1}$H} NMR Spectra of polymer obtained from ‘switch’ catalysis and polymer synthesized *via* sequential monomer addition (n. b.: the polymer from ‘switch’ catalysis used for $^{13}$C{$^{1}$H} NMR spectroscopy had a higher molar mass than the polymer obtained from sequential monomer addition). ........ 27

## List of Tables

Table S. 1 - Screening of monomers for ROCOP with [SalcyAlCl].[a] .................................... 7
Table S. 2 - Screening of monomers for ROP with [SalcyAlCl].[a] ......................................... 8
Table S. 3 – Fitting parameters based on Figure S. 2. ...................................................... 10

# 1. Materials

(±)-Propylene oxide (99%) was purchased from Sigma Aldrich and purified by fractional distillation after drying over calcium hydride overnight. Phthalic anhydride (99%, Aldrich) was purified by stirring in dry benzene overnight, filtering off insoluble impurities, evaporating of the remaining solution and recrystallization of the obtained solid in hot (anhydrous) chloroform. Sublimation under high vacuum ($10^{-2}$ mbar, 80°C) yielded phthalic anhydride, which was stored in a nitrogen-filled glovebox. *Trans*-1,2-cyclohexanediol (CHD, 98%, Aldrich) was recrystallized from anhydrous ethyl acetate, dried at 40°C under high vacuum overnight and stored inside the glovebox. (Rac)-Lactide (99%, Aldrich) was recrystallized in anhydrous toluene and sublimed under high vacuum three times. The obtained solid was stored in the glovebox at -30°C. TCA1 was synthesized according to literature procedures, recrystallized from acetone and sublimed under high vacuum before use.[1] Bis(triphenylphosphoranylidene)ammonium chloride (PPNCl, 97%, Aldrich) was recrystallized from anhydrous acetonitrile / diethyl ether, dried at 40°C overnight and stored in a glovebox. [SalphenFAlCl] was prepared according to literature procedures, dried under high vacuum for several days and stored inside the glovebox.[2] Dry toluene was obtained from a SPS-800 system by M Braun, degassed by bubbling with nitrogen for one hour and stored over activated molecular sieves. Toluene-$d_8$ and $CDCl_3$ were stirred over calcium hydride overnight, transferred under high vacuum, degassed with three freeze-pump-thaw cycles and stored over activated molecular sieves. Mesitylene (98%) was dried over calcium hydride, transferred under high vacuum, degassed by three freeze-pump-thaw cycles and stored over activated molecular sieves. All other chemicals were obtained from obtained from several commercial suppliers (Sigma Aldrich, Fischer, VWR, Alfa Aesar, Acros Organics).

# 2. Characterization

***In situ ATR-IR Spectroscopy***

For monitoring by ATR-IR, a Mettler-Toledo ReactIR 4000 spectrometer with a MCT detector and a silver halide DiComp probe was used. The reported plots were obtained after one or two point baseline corrections and the monitored bands were selected from the overlap of individual IR spectra of monomer and polymers (Figure S. 13 - Figure S. 15).

***DSC***

Differential scanning calometry (DSC) was performed on a Mettler Toledo DSC 3 Star calorimeter under nitrogen at a heating rate of 10 °C/min. First, the sample was kept at 25 °C for 1 minute, then heated to 160 °C at 10 °C/min, kept for 1 minute and cooled down to -90 °C and kept for 1 minute. This heating / cooling cycle was then repeated twice and the

data from the second or third heating cycle is reported. The flow rate of nitrogen was kept at 80 mL/min throughout the measurement.

***NMR***

$^{1}$H-NMR spectra were measured on a Bruker Avance III HD nanobay NMR equipped with a 9.4T magnet ($^{1}$H: 400.2MHz, $^{31}$P 162.0MHz) and a Bruker Avance III NMR equipped with a 11.75T magnet ($^{1}$H NMR: 500 MHz). $^{13}$C{$^{1}$H}-NMR spectra were measured on a Bruker Avance NMR equipped with a 11.75T magnet and a $^{13}$C{$^{1}$H} detect cryoprobe ($^{1}$H: 500.3MHz, $^{13}$C: 125.8MHz). All spectra were recorded in $CDCl_3$.[3]

***SEC***

Size exclusion chromatography (SEC) was performed on an Agilent PL GPC-50 instrument, with HPLC grade THF, at 30°C and a flow rate of 1.0 mL/min. In all cases, near monodisperse polystyrene standards were used for calibration. The samples were prepared by dissolving ca. 20 mg of polymer in THF, and filtering through a 2 μm PTFE filter before injection.

***End group assay with 2-chloro-4,4,5,5-tetramethyl dioxaphospholane[4]***

The procedure was previously reported by Spyros and co-workers.[4] Frist, a stock solution was prepared from bisphenol A (400 mg), $Cr(acac)_3$ (5.5 mg) and pyridine (10 mL) and stored over molecular sieves. The polymer was dissolved in $CDCl_3$ (50 mg/0.5 mL) and 40 μL of this stock solution were added before the phosphorus agent (30 μL) was added. The mixture was allowed to react for at least 30 minutes and then analysed by $^{31}$P {$^{1}$H} NMR spectroscopy after calibration with the internal standard (bisphenol A, 138.57 ppm).

## 3. Polymerisation Procedures

### Synthesis of ABA Triblock Polyesters

Inside the glovebox, [Salphen$^F$AlCl] (1 equiv., 10 mg, 16.5 µmol), PPNCl (0.9 equiv., 8 mg, 14.8 µmol), CHD (10 equiv., 19 mg, 165 µmol), PA (244 mg, 100 equiv., 1.65 mmol) and LA (237 mg, 100 equiv., 1.65 mmol) were weighed in a pre-dried vial and 1.05 mL of PO were added. The resulting suspension was sealed with PVC tape and stirred in a preheated aluminium block at 60°C for the specified time. Once full conversions were detected by $^1$H NMR spectroscopy (taken inside the glovebox), the reaction was quenched by exposure to air, excess PO was evaporated and the obtained solid was analysed by GPC. The polyester was further purified by precipitation in methanol.

### *In situ* IR Monitoring

The general polymerization procedure was followed with the following differences: First, a three neck flask suitable for the instrument was used (after drying) and the following amounts were used: PO (2.5 mL, 1000 equiv., 3.6 mmol), PA (780 mg, 100 equiv., 357 µmol), LA (950mg, 100 equiv., 357 µmol), CHD (41.5 mg, 10 equiv., 357 µmol), [SalphenFAlCl] (16.6 mg, 1 equiv., 35.7 µmol) and PPNCl (16.4 mg, 0.8 equiv., 28.6 µmol).

### *In situ* Monitoring by $^1$H NMR Spectroscopy

PO (78 µL, 150 equiv., 370 µmol), PA (55 mg, 50 equiv., 370 µmol), LA (54 mg, 50 equiv., 74 µmol), CHD (9 mg, 10 equiv., 74 µmol), [Salphen$^F$AlCl] (3.5 mg, 1.0 equiv., 7.4 µmol), PPNCl (3 mg, 0.8 equiv., 5.9 µmol) and mesitylene (10 µL, 10 equiv., 74 µmol) were added to a Young's Tap NMR tube with 0.5 mL of $CDCl_3$ inside the glovebox, sealed, and added to a 500 MHz NMR instrument preheated to 60°C. Every 120 seconds, a $^1$H NMR spectrum was recorded (number of scans=8; number of dummy scans=0). In total, 435 NMR spectra were collected, which were processed in Mestrenova (automatic baseline correction and phasing) after normalising against mesitylene.

### Synthesis of Multiblock Polyesters

The general procedure outlined above was followed after mesitylene (10 equiv.) were added to reaction mixture. After each monomer addition, the reaction was left to react for at least 20 hours before aliquots were taken inside the glovebox and conversions were analysed by $^1$H NMR spectroscopy and GPC. It should be noted that the solution became highly viscous, particularly at high block numbers, and additional PO (1 mL) was added, the reaction was transferred to a larger vial and a rare-earth stirring bar was used. The final polymer was purified as described above.

## 4. Illustration of Possible Polymeric Products From Mixed Feedstocks Containing Anhydrides, Epoxides and Lactones

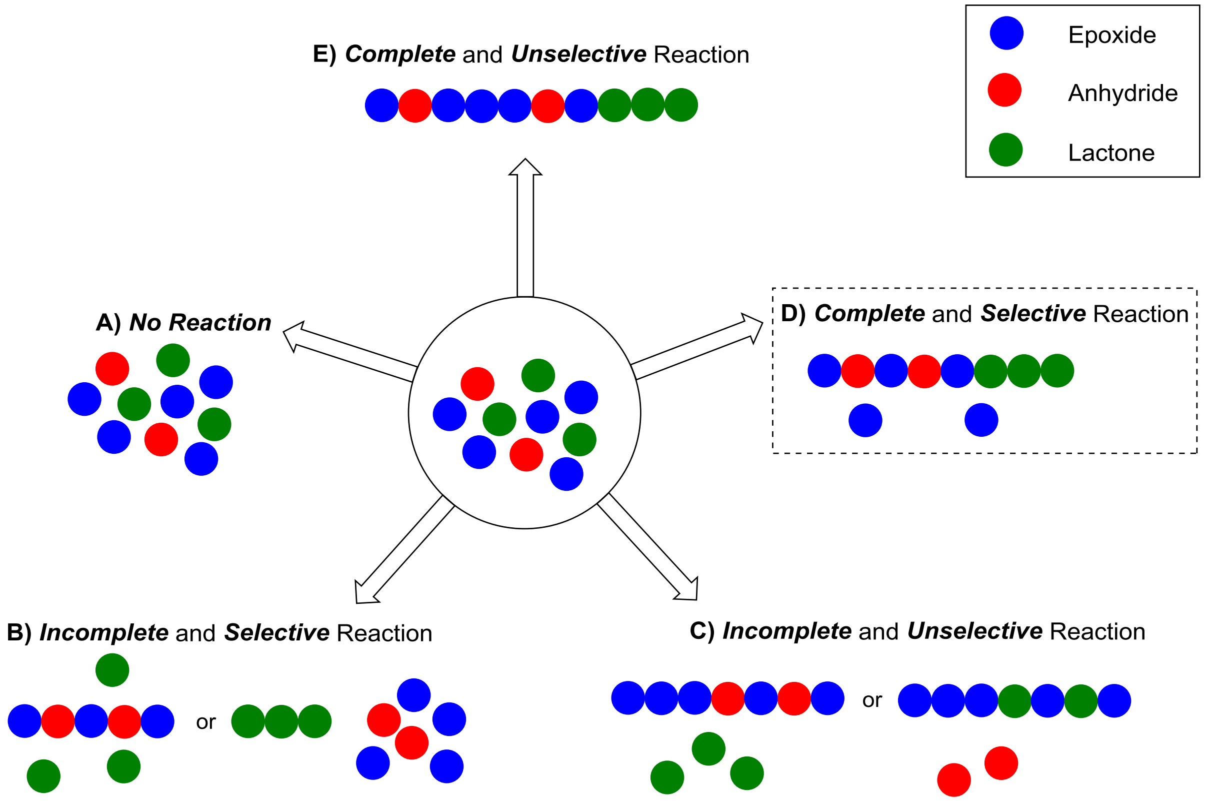


Scheme S. 1 – Possible outcomes for a Polymerisation of a mixed feedstock containing three monomers.

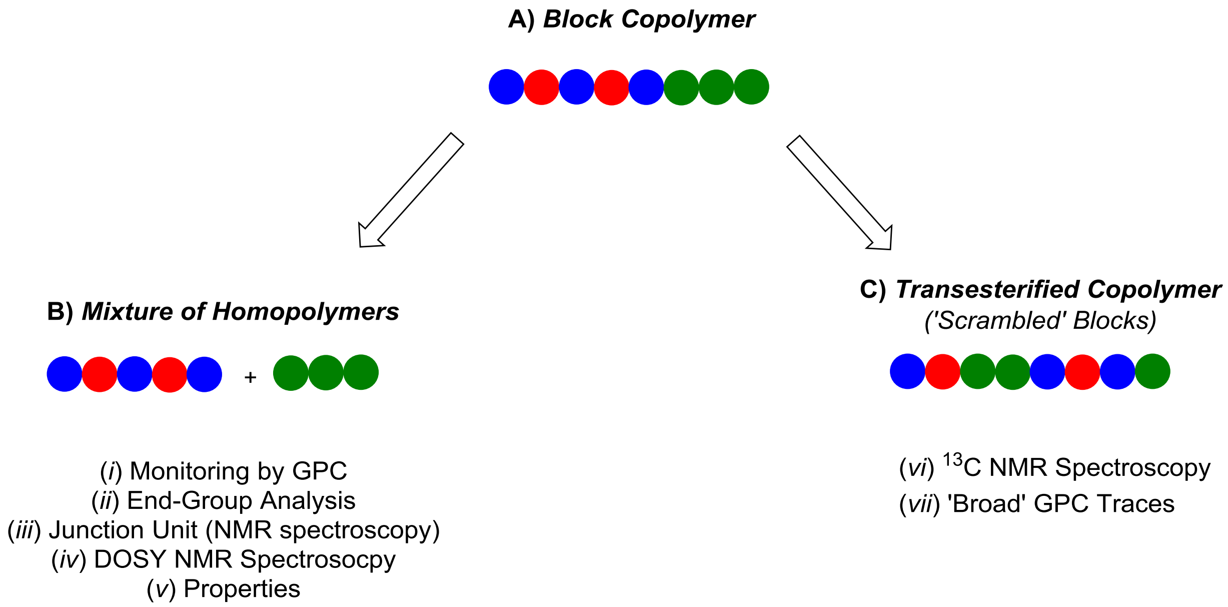


Scheme S. 2 - Possible materials in 'switch' catalysis and recommendations of how to distinguish between them.[5]

## 5. Initial Screening with [SalcyAlCl]

Table S. 1 - Screening of monomers for ROCOP with [SalcyAlCl].[a]

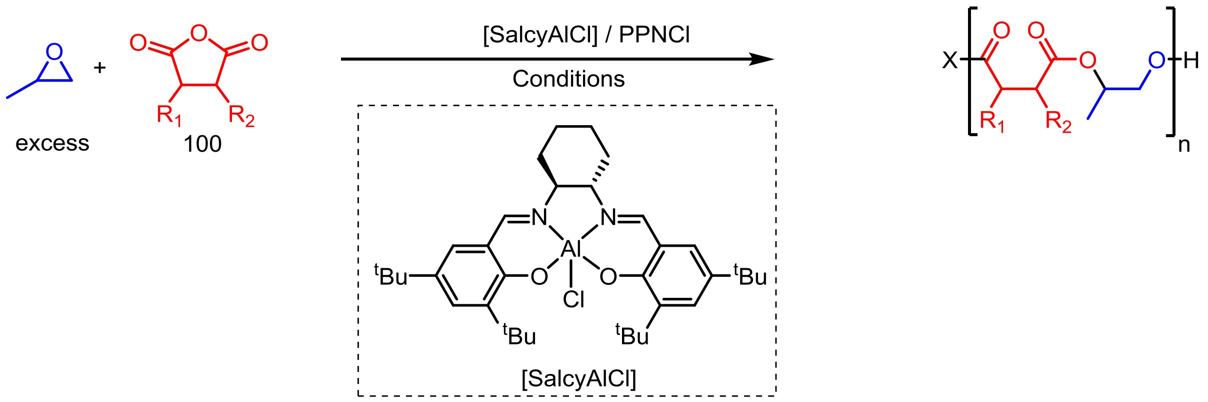


| Anhydride | t [d] | Conversion [%][b] | Selectivity [%][b] | $M_n$ ($Đ$) [g mol$^{-1}$][c] |
|---|---|---|---|---|
| 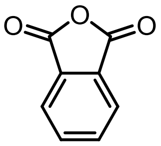 PA | 2 | > 95 % | > 95 % | 18,100 (1.77) |

[a] Conditions: [Catalyst]: [Co-catalyst]: [Anhydride]: [Epoxide] = 1:0.9:100:1000, room temperature. [b] Determined by $^1$H NMR spectroscopy. [c] Determined by GPC (THF, 30°C), based on PS standards.

Table S. 2 - Screening of monomers for ROP with [SalcyAlCl].[a]

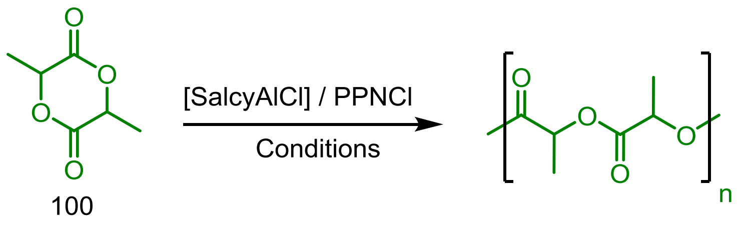


| Lactone | t [d] | Conversion [%][b] | Selectivity [%][b] | $M_n$ (*Đ*) [g mol$^{-1}$][c] |
|---|---|---|---|---|
| 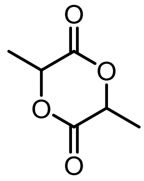 LA | 1 | 99 | > 95 % | 8,700 (1.40) |

[a] Conditions: [Catalyst]:[Co-catalyst]:[Lactone]:[Epoxide] = 1:0.9:100:1000, room temperature. [b] Determined by $^1$H NMR spectroscopy. [c] Determined by GPC (THF, 30°C), based on PS standards.

## 6. One-Pot Polymerisation of PO/PA/LA with [Salphen$^F$AlCl]

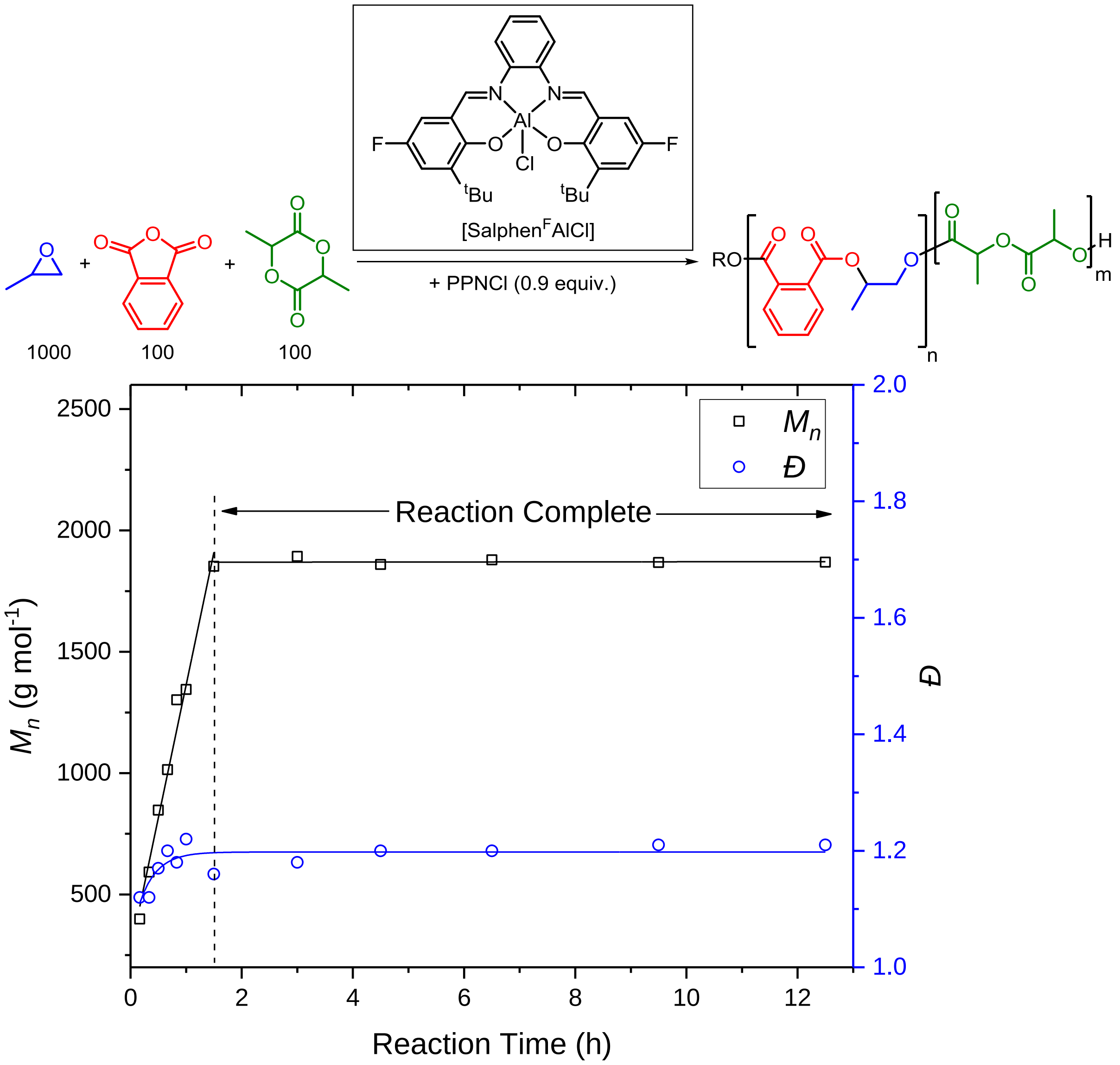


Figure S. 1 – One-pot polymerisation of PO/PA/Lactide (top) and GPC analysis at different times (bottom). Conditions: [Salphen$^F$AlCl]:[PPNCl]:[CHD]:[PA]:[LA]:[PO] = 1:0.8:10:100:100:1000, 60°C.

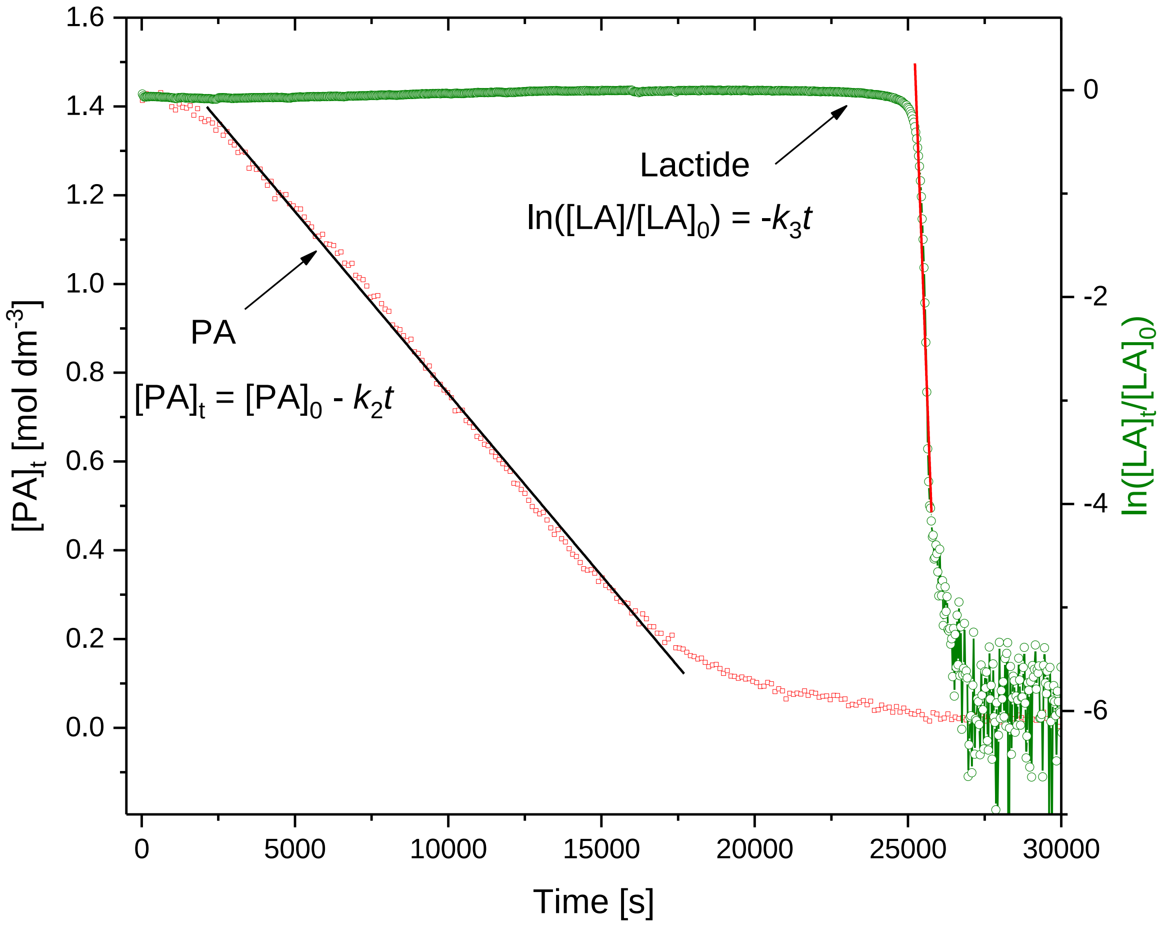


Figure S. 2 – Kinetics analysis of in situ IR plot shown in Fig. 1.

Table S. 3 – Fitting parameters based on Figure S. 2.

| | | |
|---|---|---|
| Equation | y = a + b*x | y = a + b*x |
| Monomer | PA | LA |
| Weight | No Weighting | No Weighting |
| Intercept | 208.20656 ± 10.55889 | 1.57331 ± 0.00208 |
| Slope | -0.00824 ± 4.13404E-4 | -8.20182E-5 ± 1.90659E-7 |
| Residual Sum of Squares | 1.84198 | 0.19857 |
| Pearson's r | -0.97808 | -0.9986 |
| R-Square(COD) | 0.95665 | 0.99721 |
| Adj. R-Square | 0.95424 | 0.9972 |

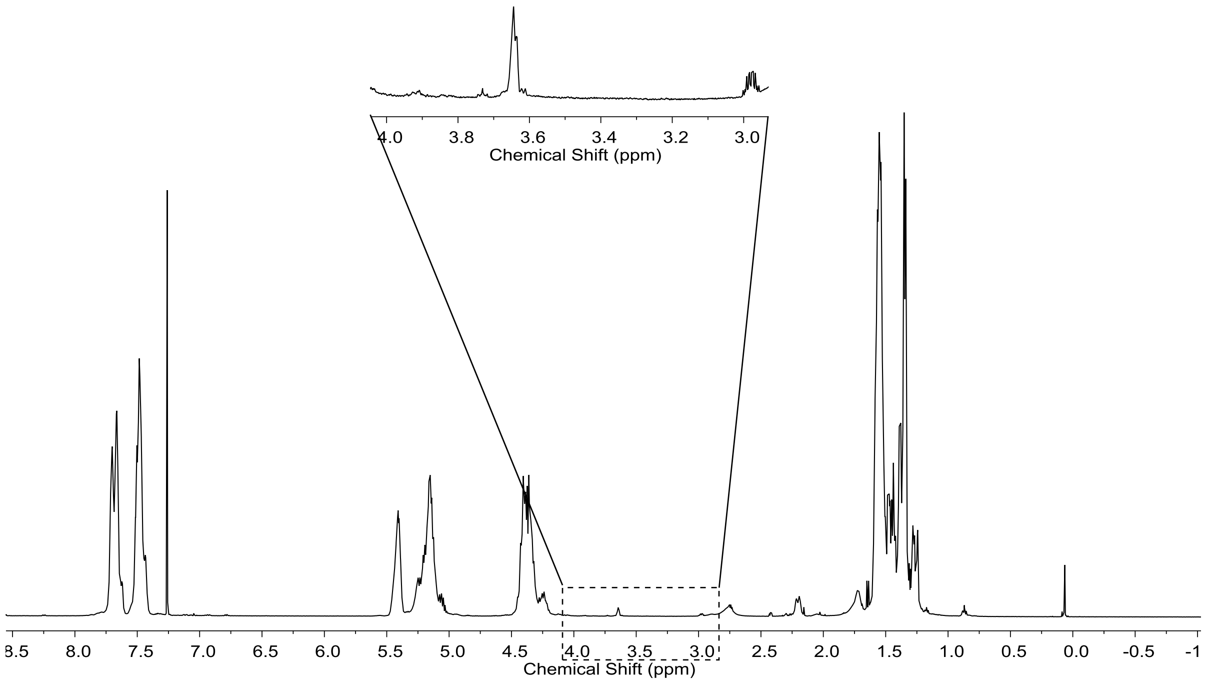


Figure S. 3 – $^1$H NNR spectrum of isolated polyester; the region relevant to the formation of polyether between 3.0 and 4.0 ppm is enlarged (polyether should be observed as broad resonances in this area).

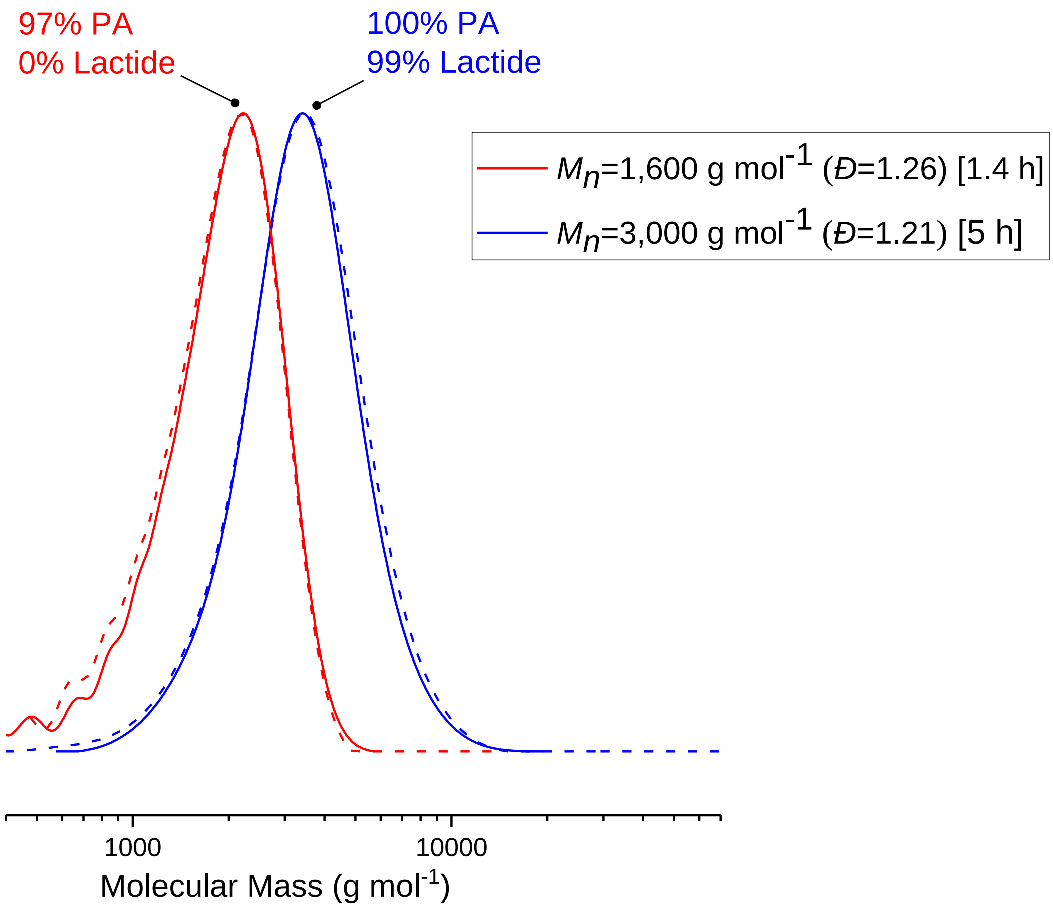


Figure S. 4 - GPC traces of one-pot polymerisation of PO/PA/LA with [Salphen$^F$AlCl]/PPNCl. RI trace is given as a solid, UV trace as a dashed line. Conditions: [Salphen$^F$AlCl]:[PPNCl]:[CHD]:[PA]:[LA]:[PO] = 1:0.8:10:100:100:1000, 60°C.

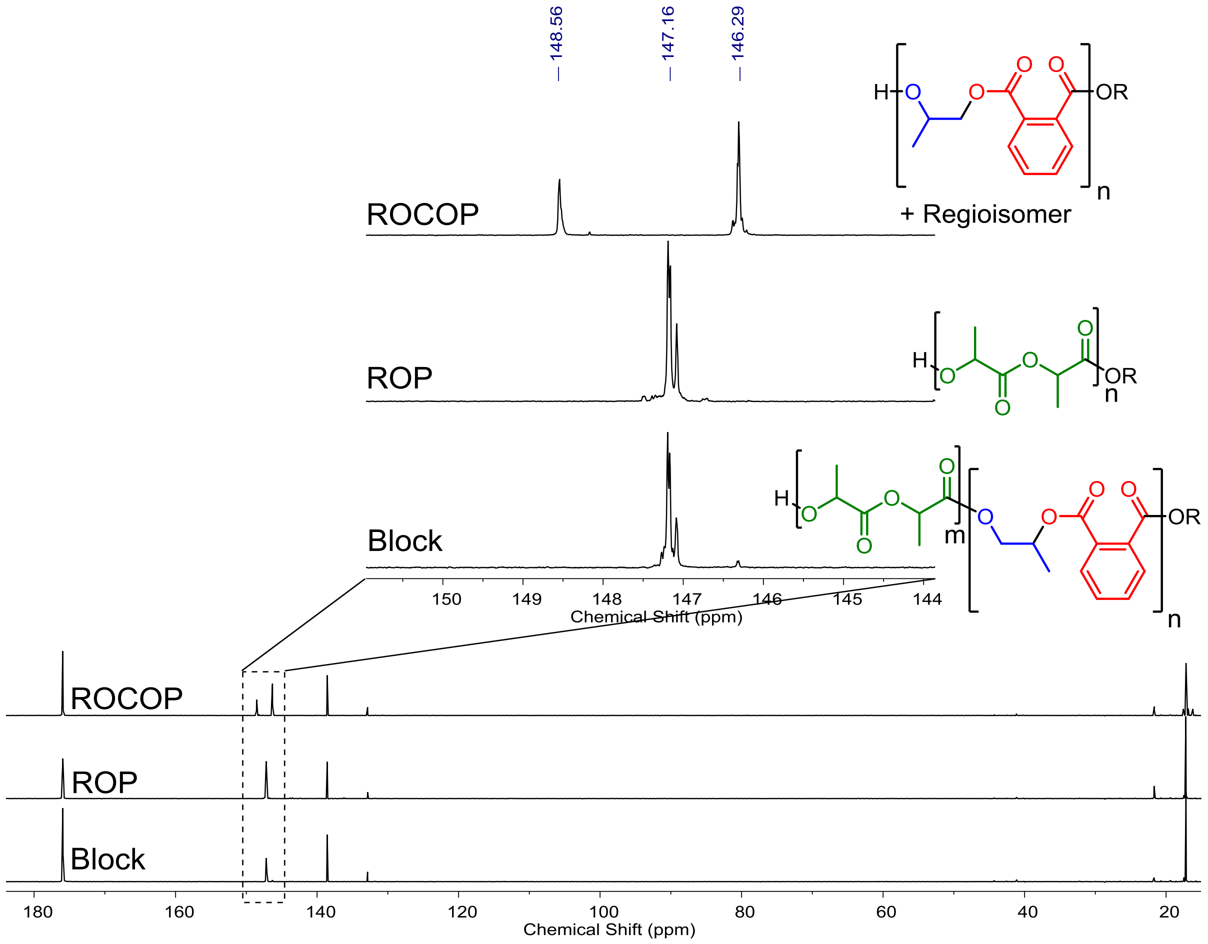


Figure S. 5 - $^{31}P\{^{1}H\}$ NMR spectra of different polyesters, after the reaction with 2-chloro-4,4,5,5-tetramethyl-1,3,2-dioxaphospholane (bisphenol A present as internal standard).

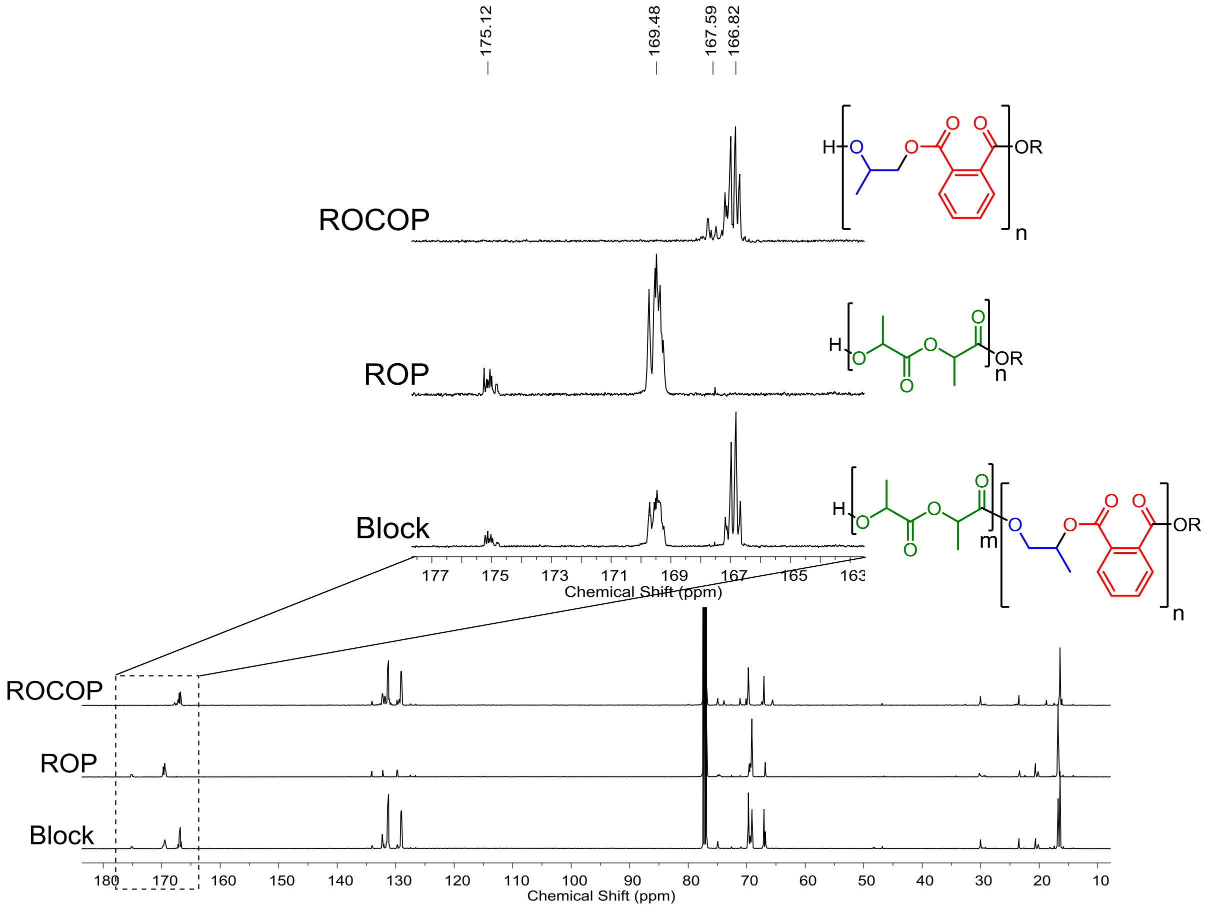


Figure S. 6 - Comparison of the $^{13}C\{^{1}H\}$ NMR spectra of the block copolymer and the separate polymers obtained from ROP or ROCOP.

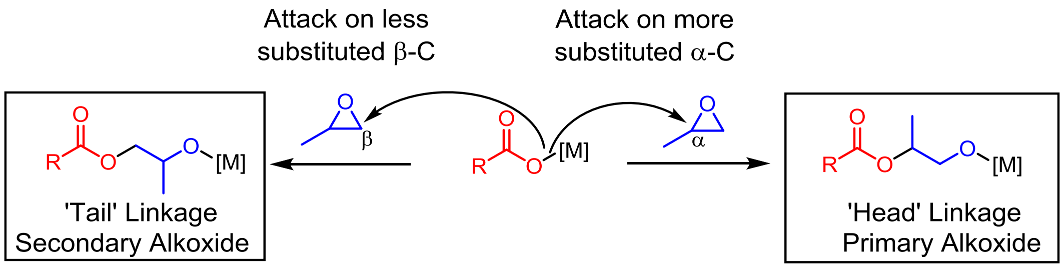


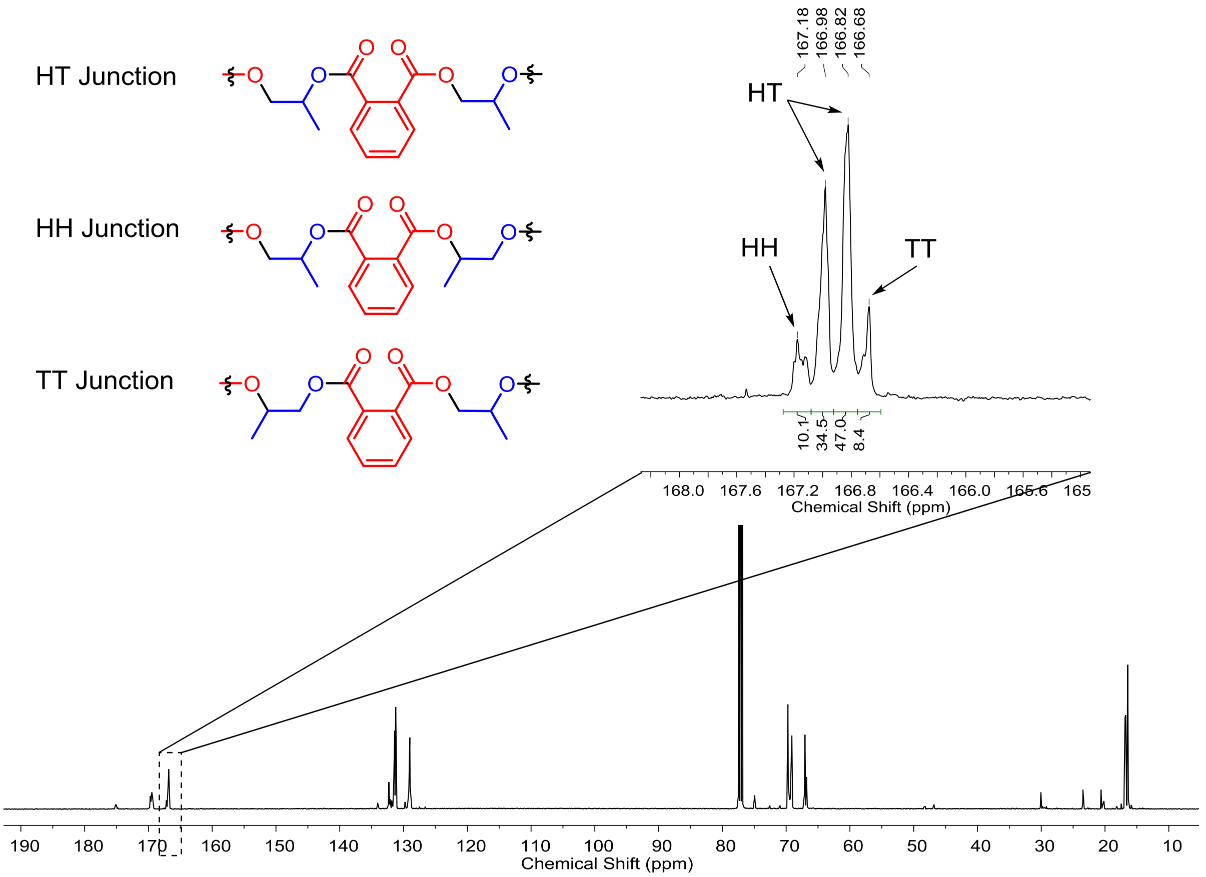


Figure S. 7 - $^{13}C\{^{1}H\}$ NMR spectrum of isolated block copolyester and an illustration of regioselective epoxide ring-opening.

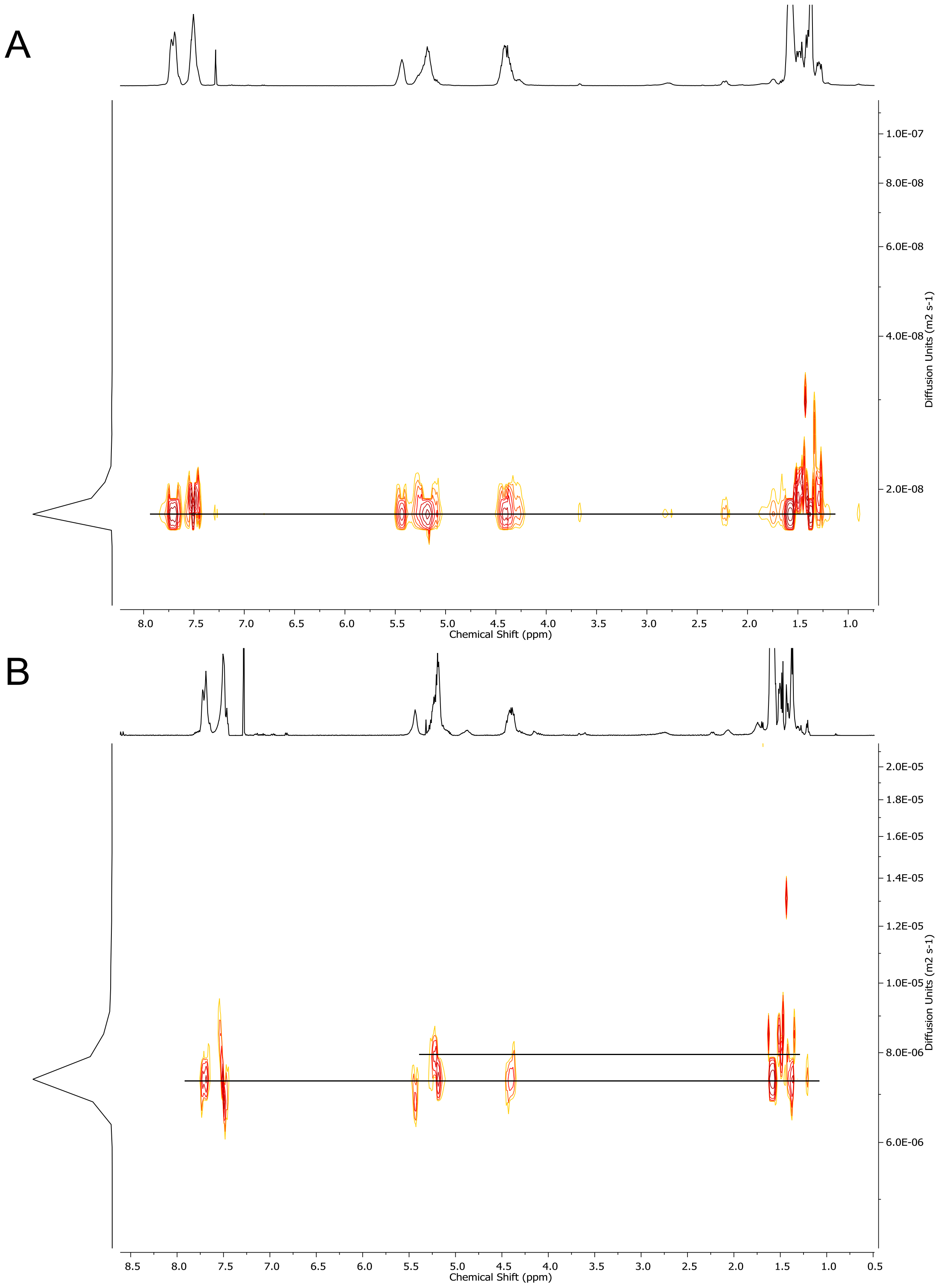


Figure S. 8 - DOSY NMR Spectra of block copolymer (A) and blend of homopolymers (B).

## 7. $^1$H NMR Spectra of Multiblock Copolyesters

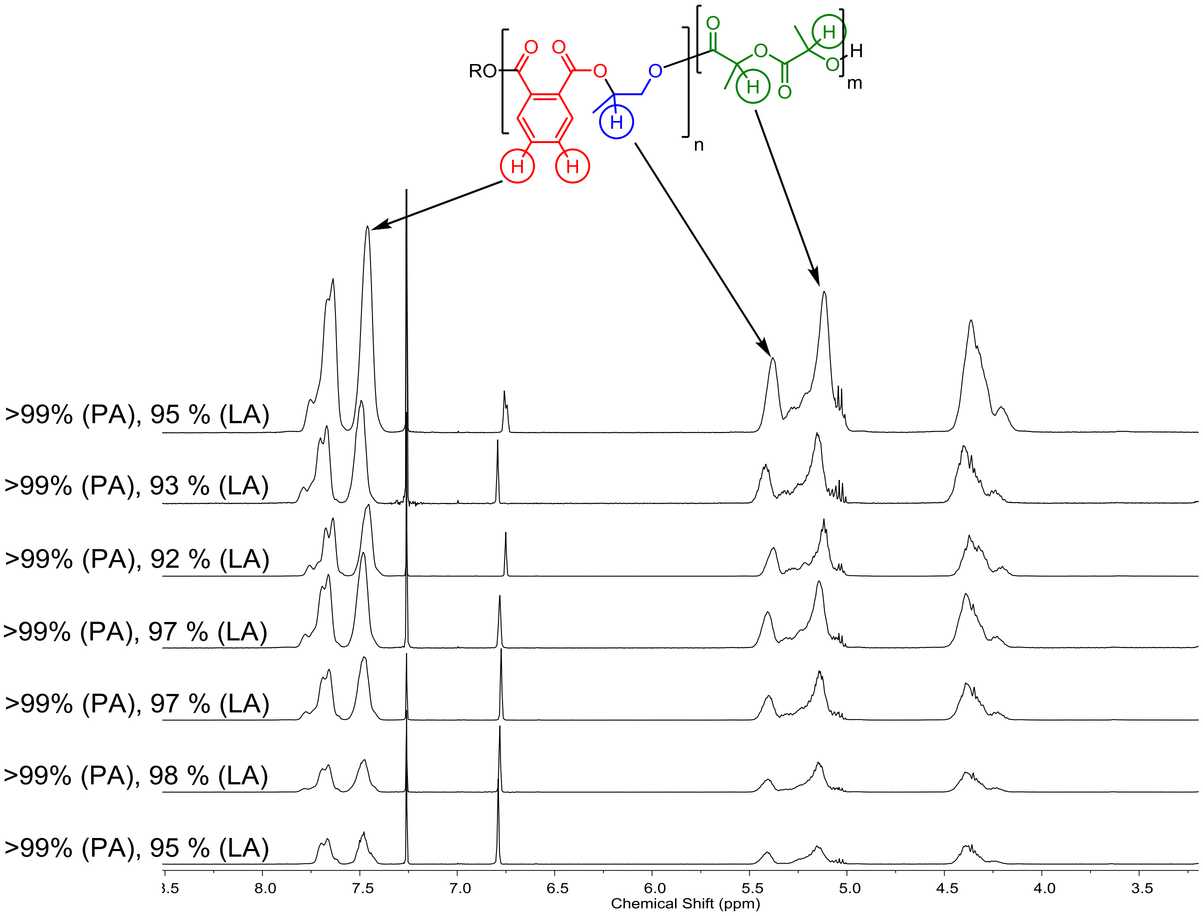


Figure S. 9 - $^1$H NMR spectra of crude reaction mixture after each monomer addition; typical reaction times: 6 hours – 2 days. Conversions are based on mesitylene (6.80 ppm) as internal standard.

## 8. One-Pot Polymerisation of PO/TAC1/LA with [Salphen$^F$AlCl]

Inside the glovebox, [Salphen$^F$AlCl] (1 equiv., 1 mg, 1.6 μmol; from a stock solution of 10 mg/1.5 mL of PO), PPNCl (0.9 equiv., 1 mg, 1.48 μmol; from a stock solution of 50mg/250μL acetonitrile), CHD (20 equiv., 5 mg, 32 μmol), TAC1 (1.0 g, 2,000 equiv., 3.2 mmol) and LA (618 mg, 2,000 equiv., 3.2 mmol) were weighed in a pre-dried vial and 1.5 mL of PO were added. The resulting suspension was sealed with PVC tape and stirred in a preheated aluminium block for 7 seven days. The final polyester was purified by precipitation from methanol.

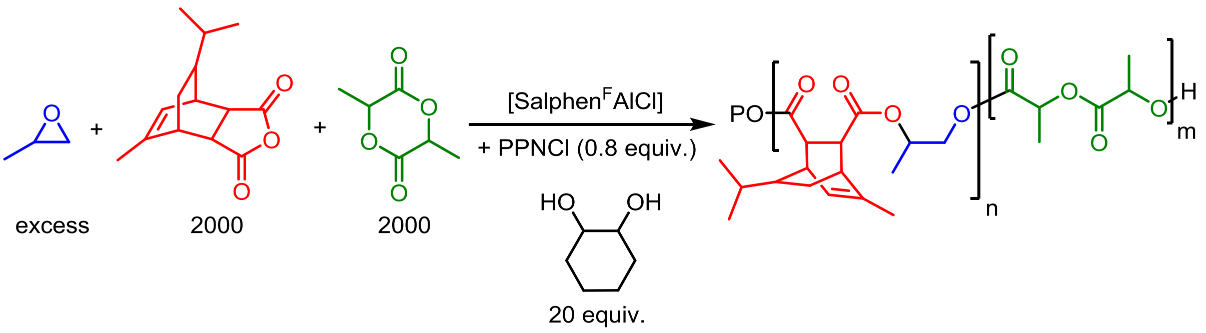


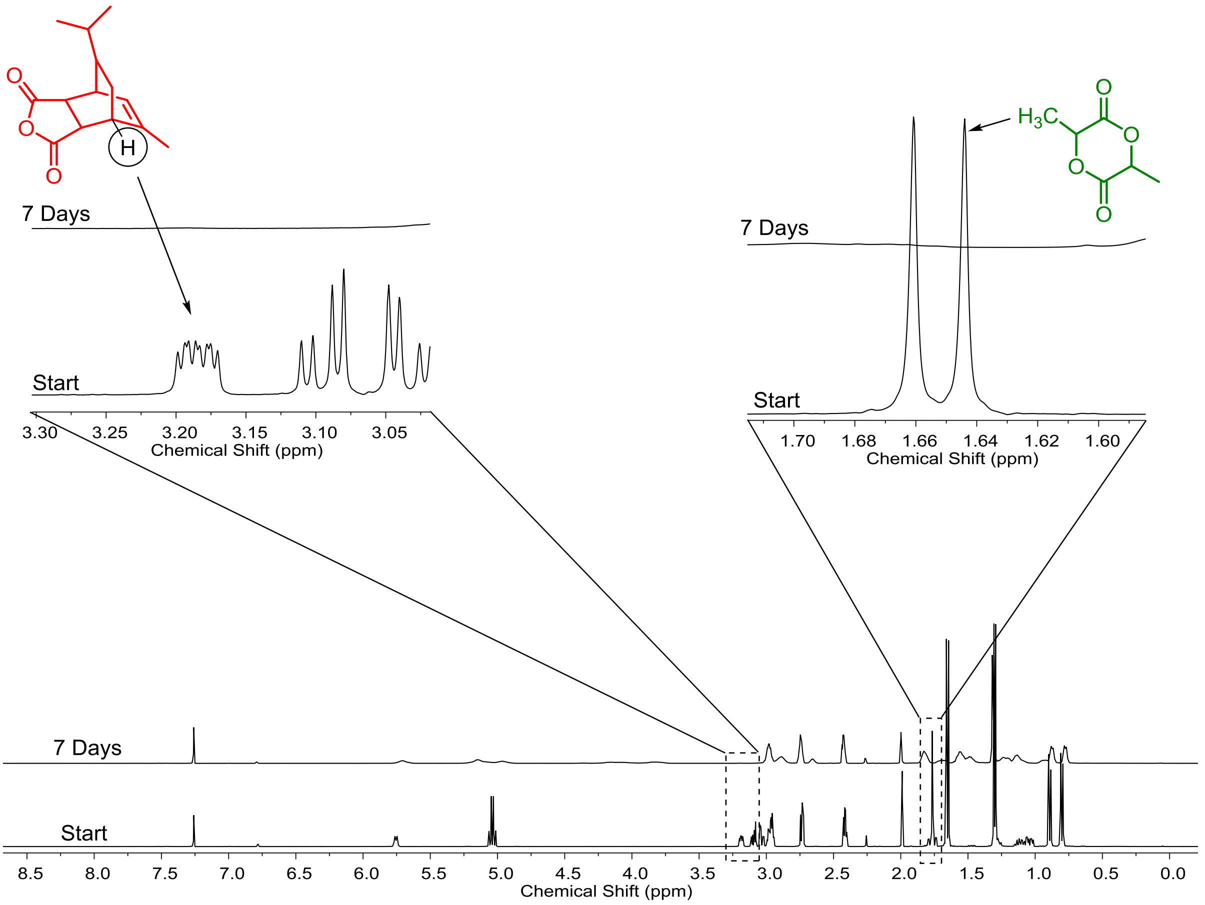


Figure S. 10 – $^1$H NMR spectrum of the one-pot polymerisation of TCA1/PO/LA

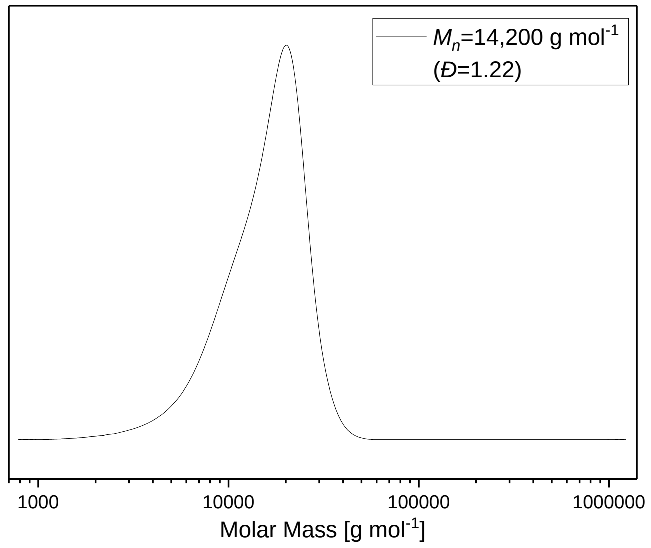


Figure S. 11 – GPC trace of polymer obtained from TCA1/PO/LA.

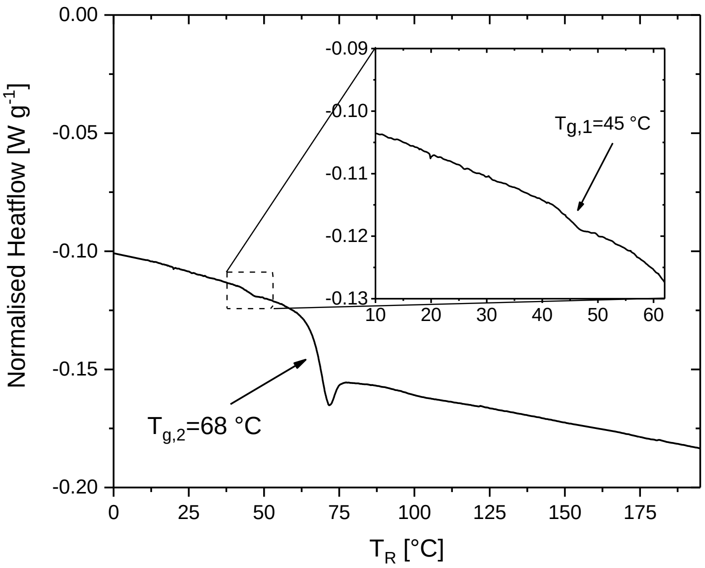


Figure S. 12 – DSC Thermogram of purified polymer based on PO/PA/LA.

## 9. IR Spectra of Monomers and Polymers

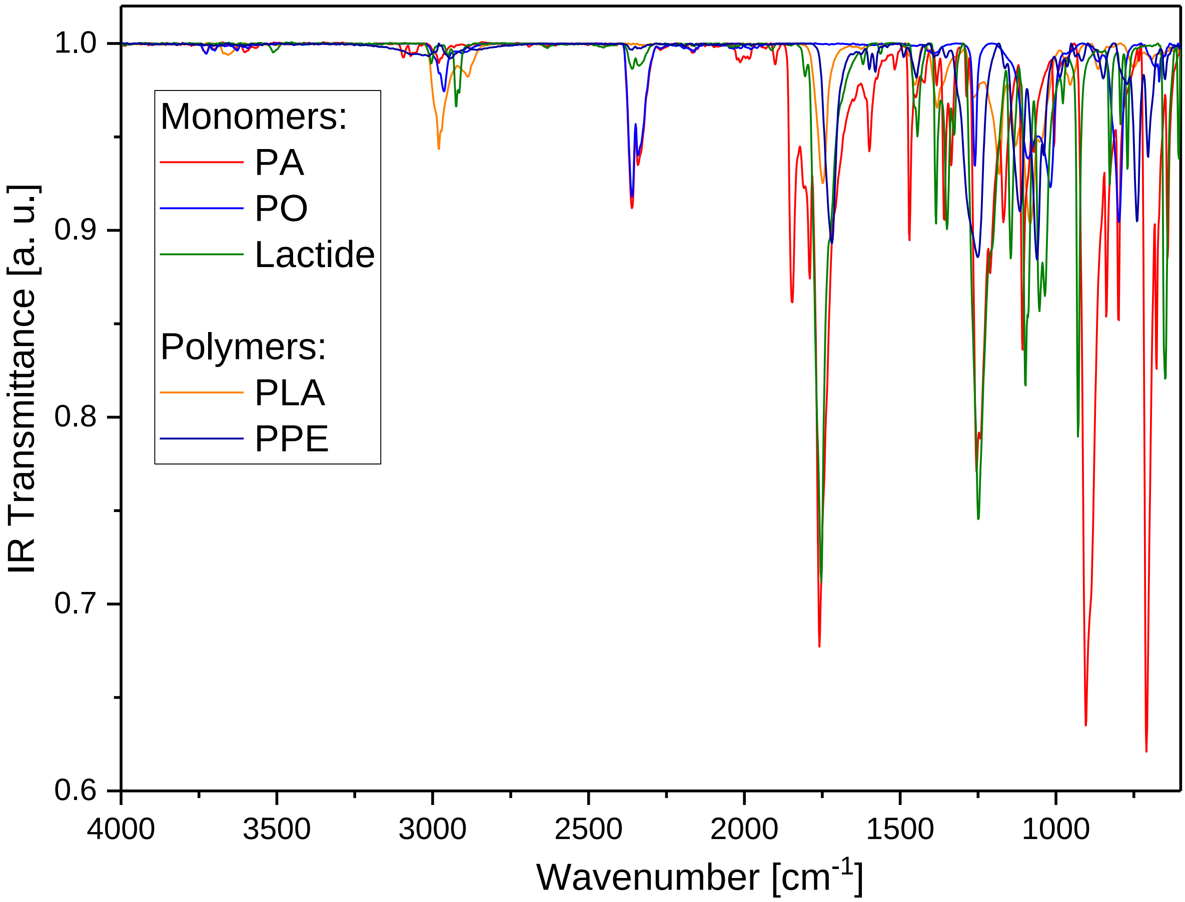


Figure S. 13 – IR spectra of monomers and polymers.

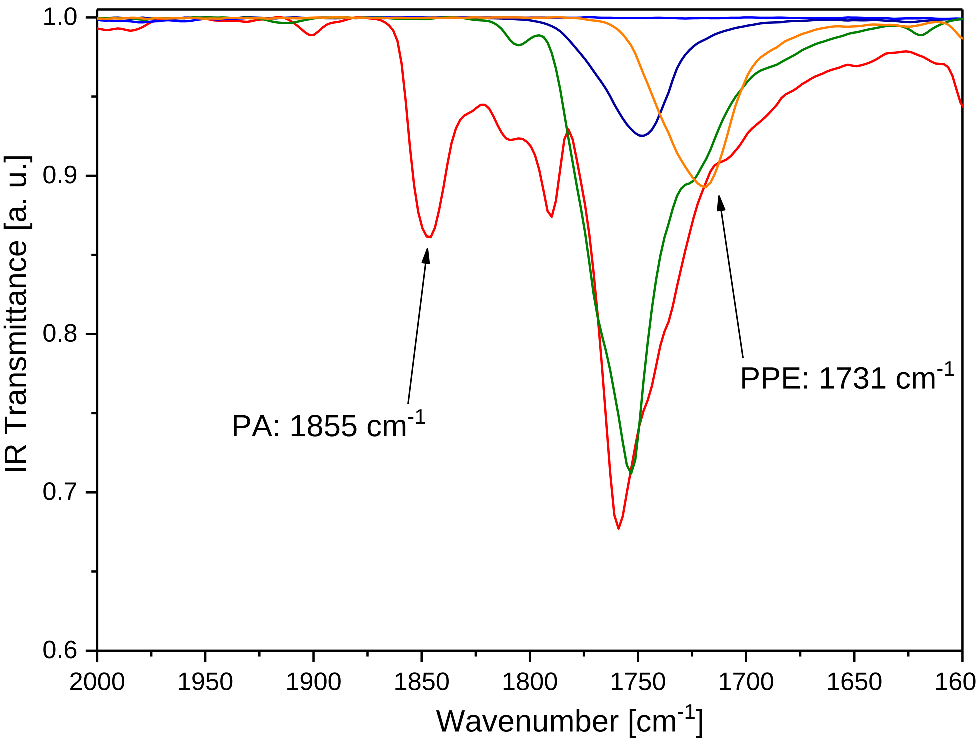


Figure S. 14 – Extended region of IR spectra for PA and PPE.

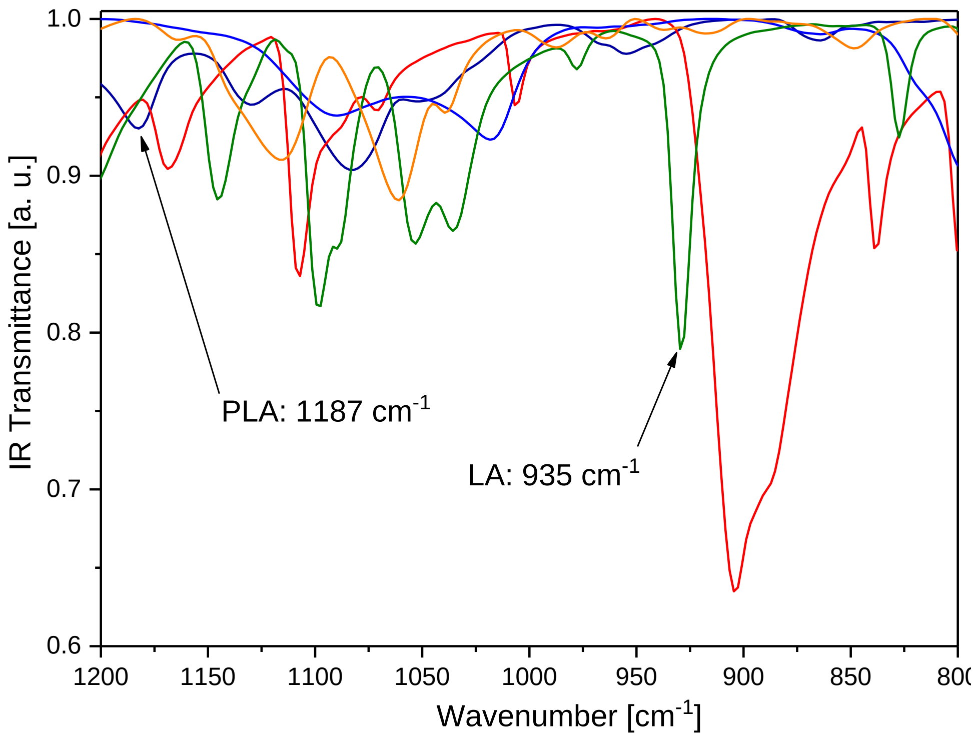


Figure S. 15 – Extended region of IR spectra for LA and PLA.

## 10. ¹H NMR Spectra of PLA-b-PPE-b-PLA

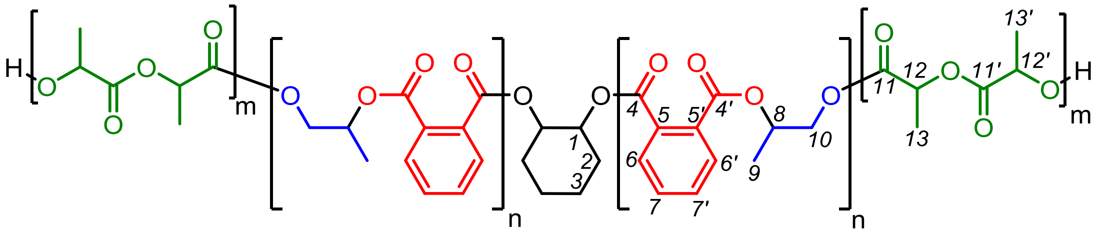

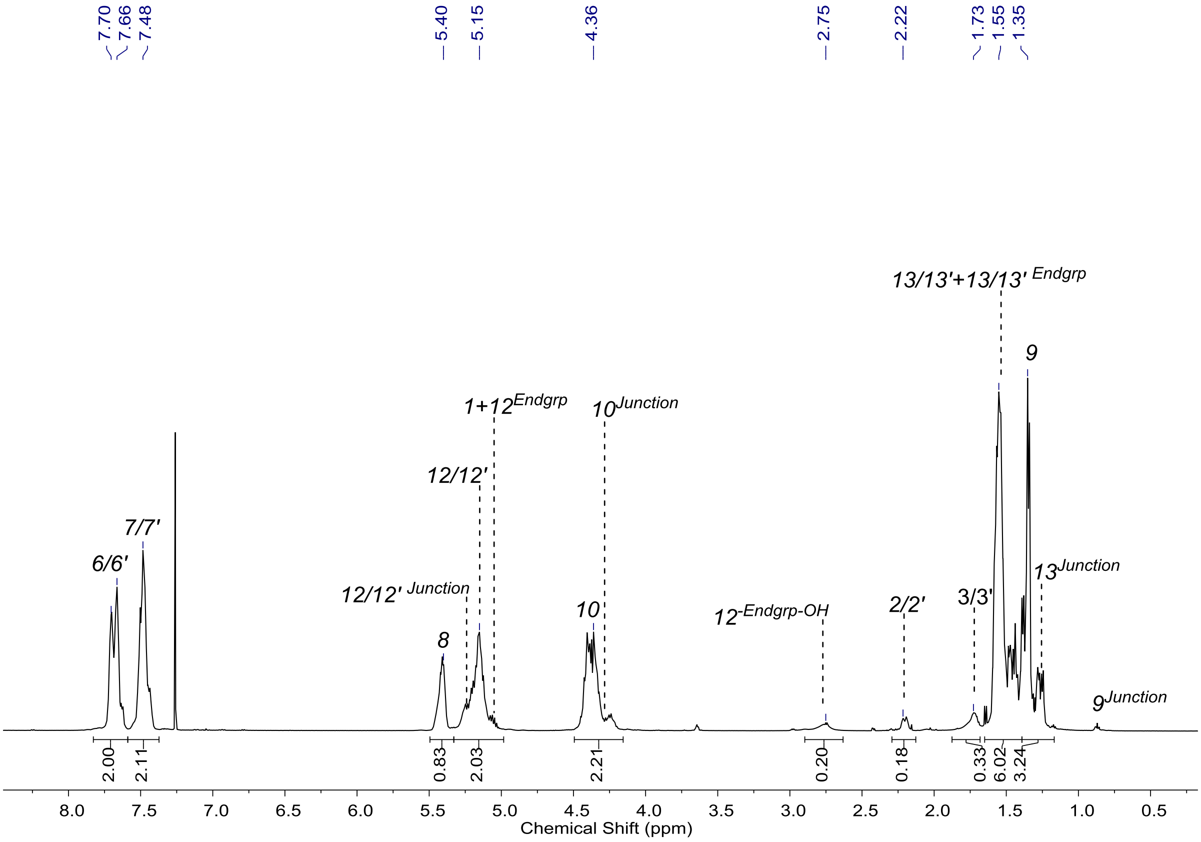


Figure S. 16 – ¹H NMR spectrum of isolated polymer.

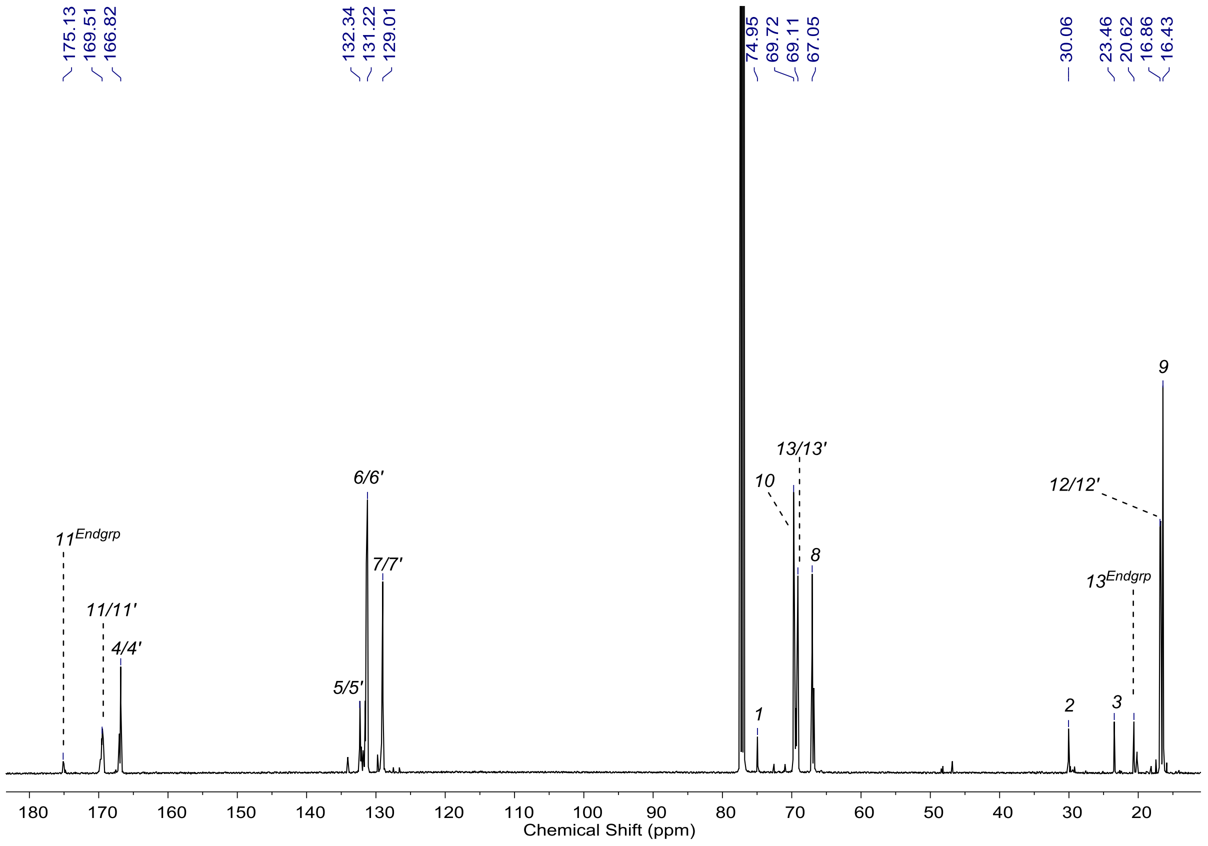


Figure S. 17 – $^{13}C\{^{1}H\}$ NMR spectrum of isolated polymer.

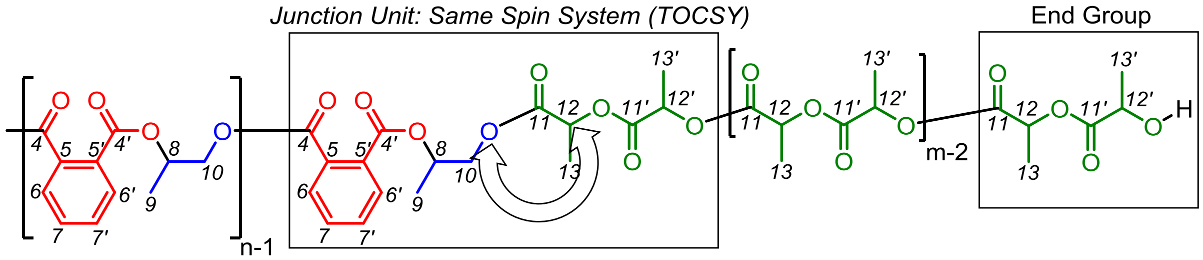


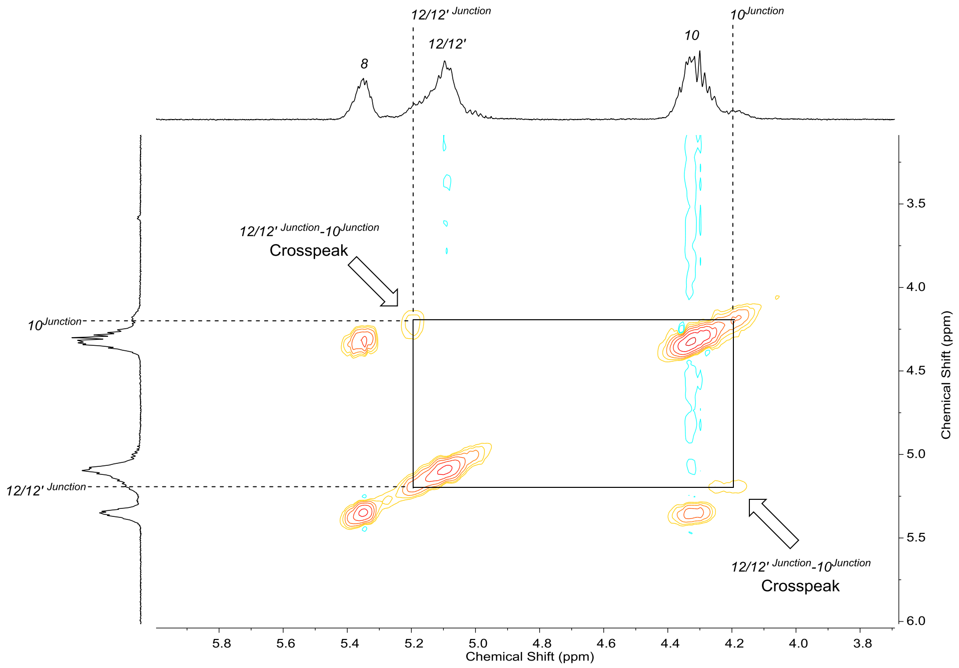


Figure S. 18 – $^{1}H$,$^{1}H$ TOCSY NMR spectrum (only region relevant to junction unit shown).

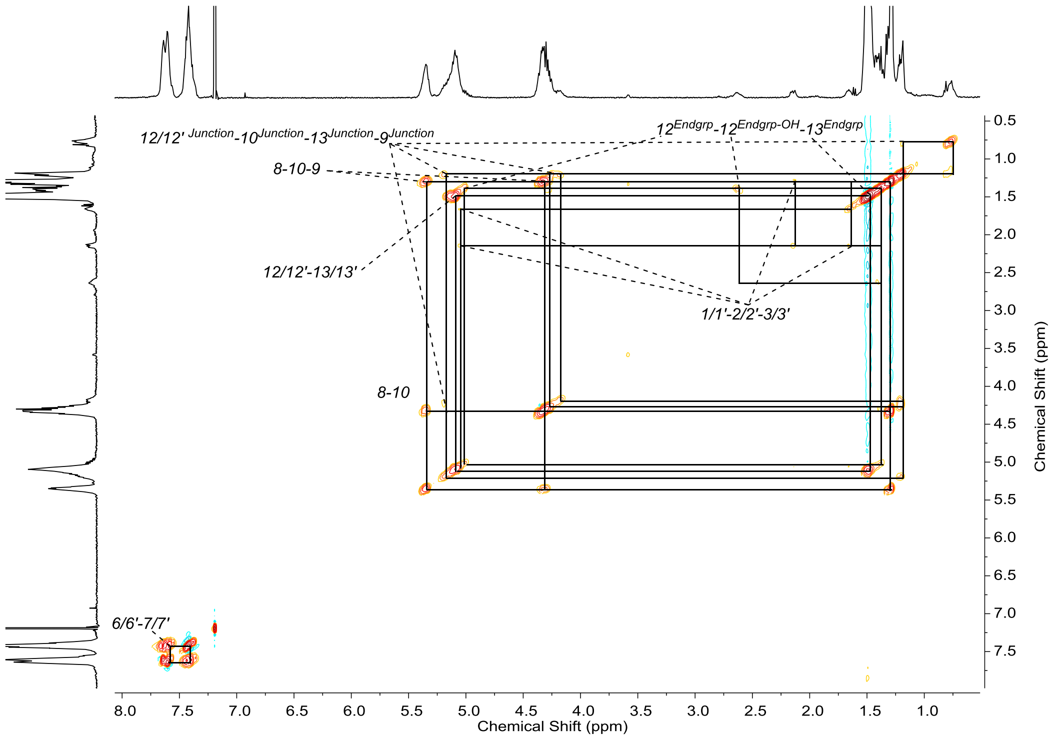


Figure S. 19 – $^{1}H$,$^{1}H$ TOCSY NMR spectrum.

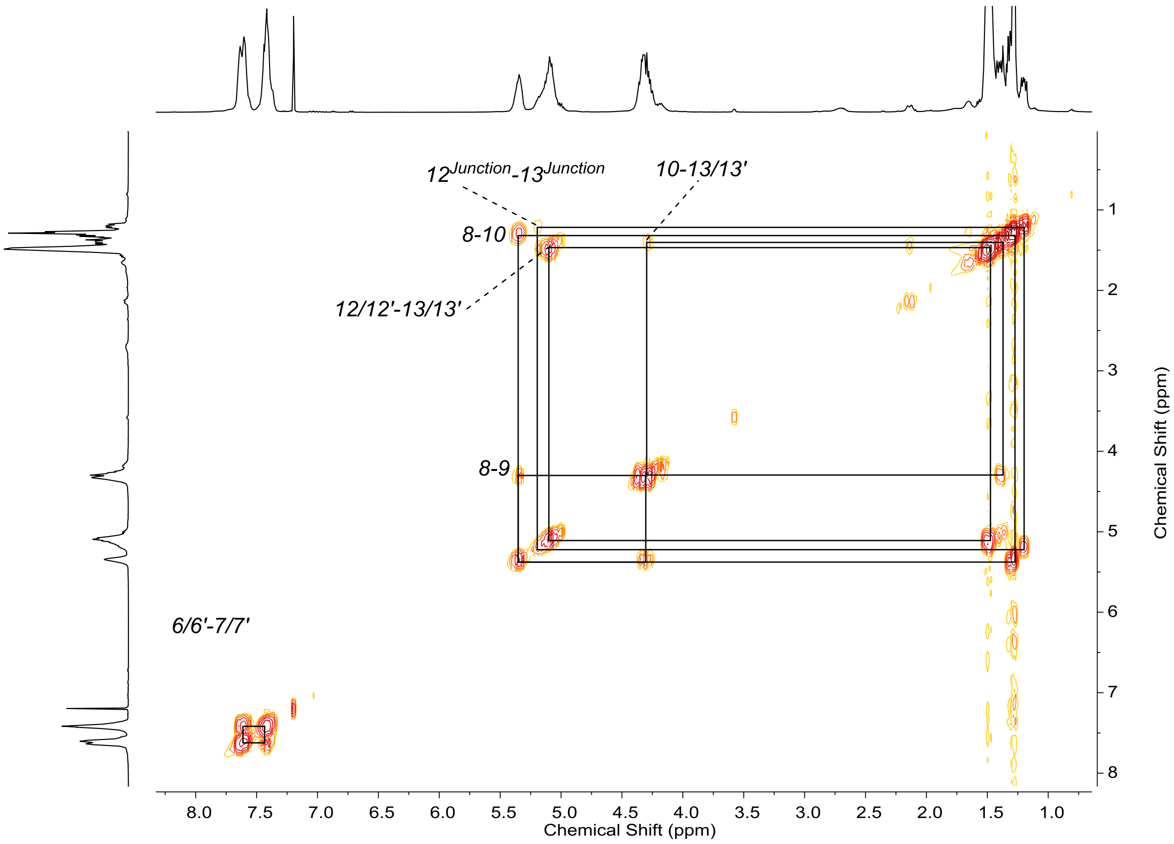


Figure S. 20 – $^{1}H,^{1}H$ COSY NMR Spectrum of PLA-*b*-PPE-*b*-PLA.

# 11. Polyester obtained from sequential Monomer Addition

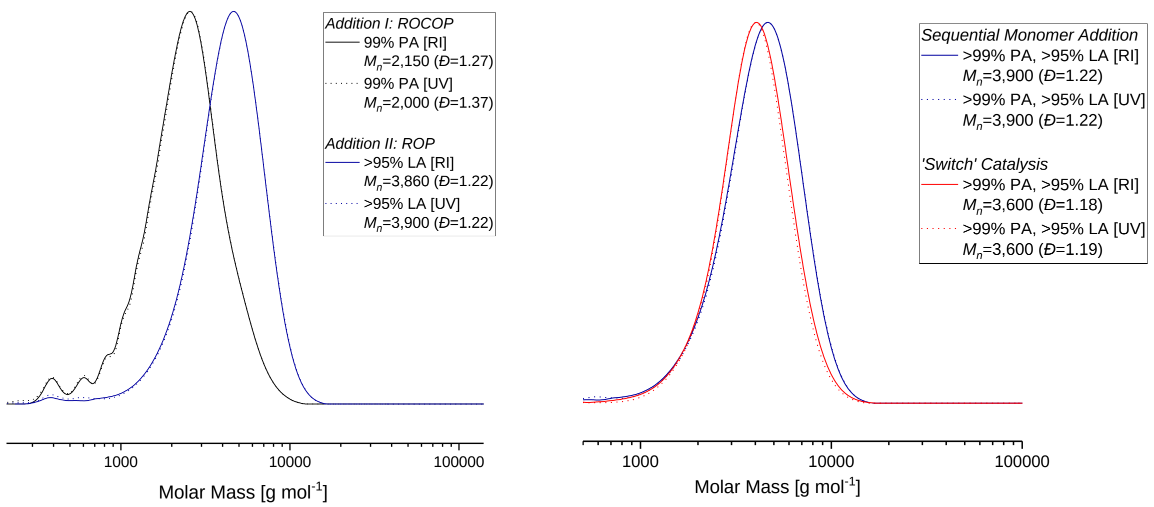


Figure S. 21 – GPC Traces of block polyester formed through sequential monomer addition (left) and comparison between polyesters obtained from sequential monomer addition and ‘switch’ catalysis.

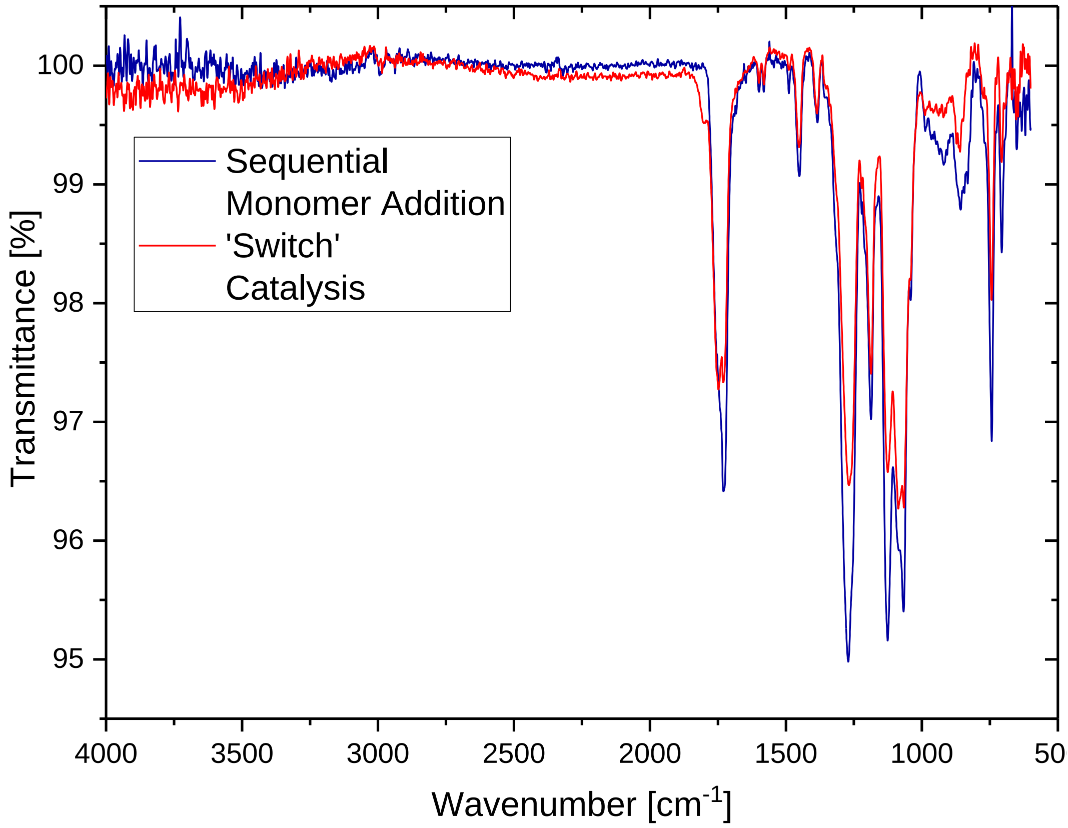


Figure S. 22 – IR Spectra of polymer obtained from ‘switch’ catalysis and polymer synthesized *via* sequential monomer addition.

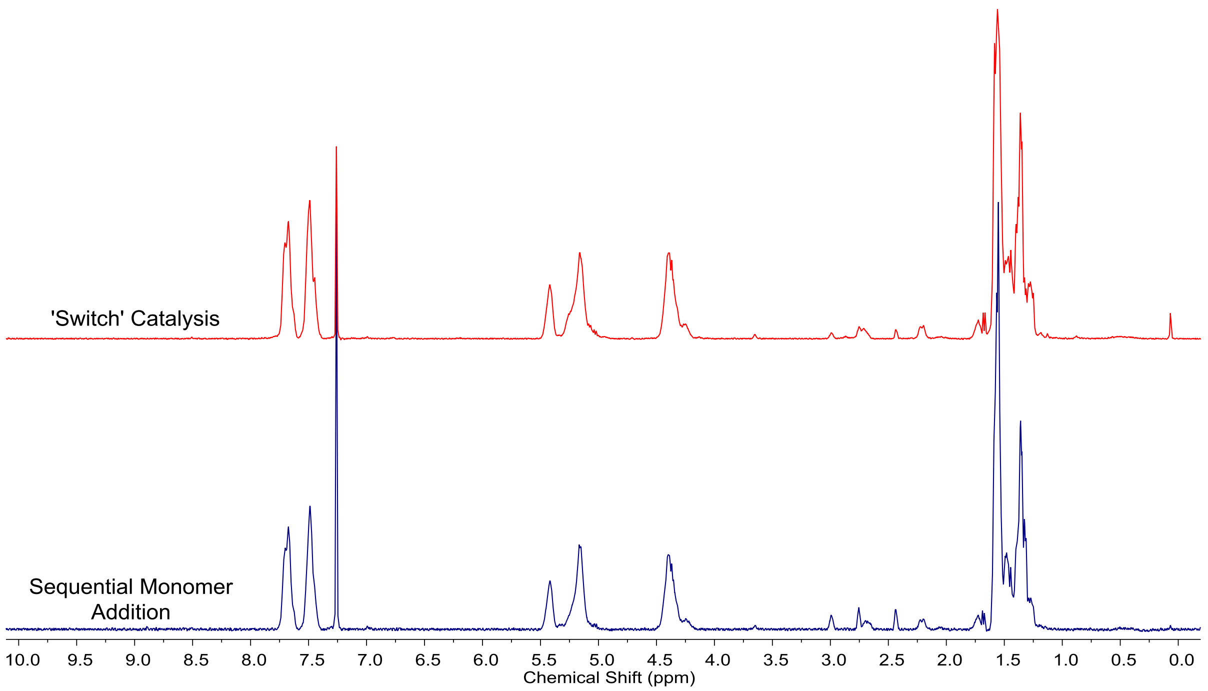


Figure S. 23 – $^1$H NMR Spectra of polymer obtained from 'switch' catalysis and polymer synthesized *via* sequential monomer addition.

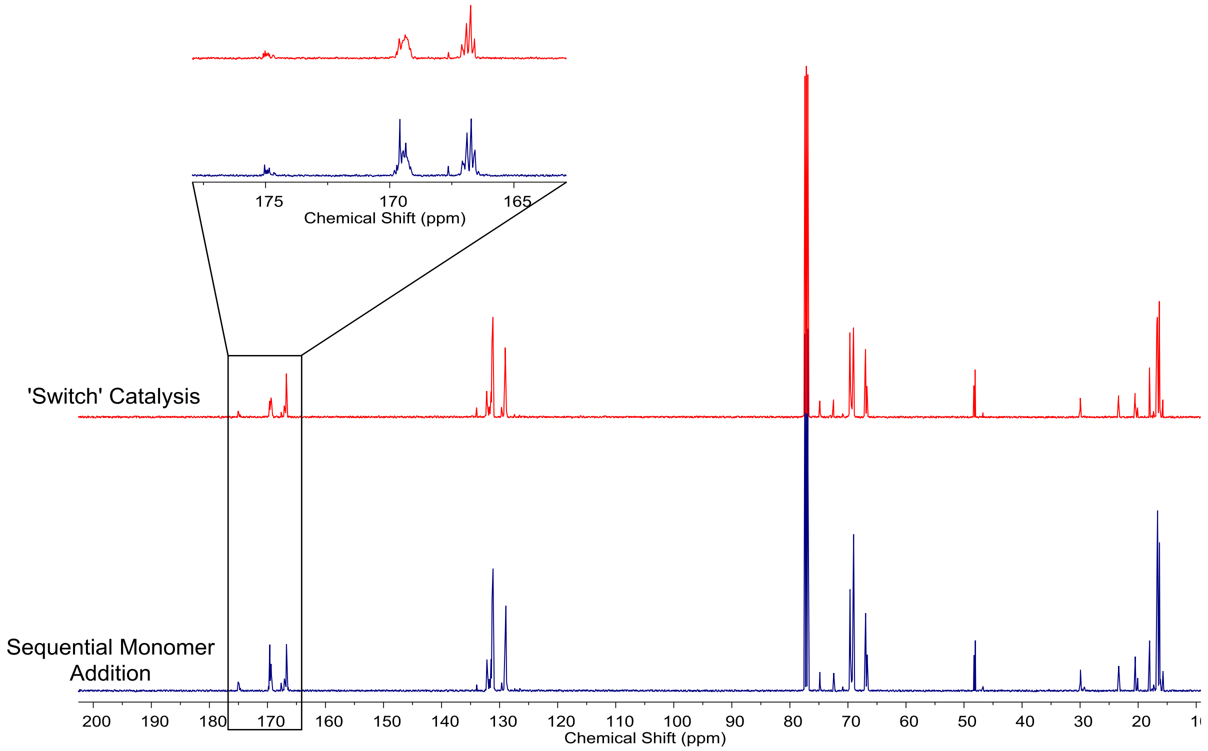


Figure S. 24 – $^{13}$C{$^1$H} NMR Spectra of polymer obtained from 'switch' catalysis and polymer synthesized *via* sequential monomer addition. The carbonyl region is enlarged.

## 12. References


[1] D. Dakshinamoorthy, A. K. Weinstock, K. Damodaran, D. F. Iwig, R. T. Mathers, *ChemSusChem* **2014**, *7*, 2923-2929.

[2] N. J. Van Zee, M. J. Sanford, G. W. Coates, *J. Am. Chem. Soc.* **2016**, *138*, 2755-2761.

[3] G. R. Fulmer, A. J. M. Miller, N. H. Sherden, H. E. Gottlieb, A. Nudelman, B. M. Stoltz, J. E. Bercaw, K. I. Goldberg, *Organometallics* **2010**, *29*, 2176-2179.

[4] A. Spyros, D. S. Argyropoulos, R. H. Marchessault, *Macromolecules* **1997**, *30*, 327-329.

[5] T. Stößer, T. T. D. Chen, Y. Zhu, C. K. Williams, *Phil. Trans. R. Soc. A* **2018**, *376*, 20170066.
